# Artificial intelligence-driven identification and mechanistic exploration of synergistic anti-aging compounds from Dengzhan Shengmai formulation

**DOI:** 10.1371/journal.pone.0356888

**Published:** 2026-09-08

**Authors:** Jingyi Hou, Xueli Li, Miao Gu, Kaikai Ding, Kailan Yang, Bowen Xu

**Affiliations:** 1 Hebei Province Key Laboratory of Study and Exploitation of Chinese Medicine, Chengde Medical University, Chengde, China; 2 Chengde Medical University, Chengde, China; 3 Beijing Key Laboratory of Traditional Chinese Medicine Basic Research on Prevention and Treatment for Major Diseases, Experimental Research Center, China Academy of Chinese Medical Sciences, Beijing, China; 4 Hebei Key Laboratory of Nerve Injury and Repair, Chengde Medical University, Chengde, Hebei, China; Foshan University, CHINA

## Abstract

Aging is a complex biological process involving multiple dysregulated pathways, and synergistic compound combinations offer distinct therapeutic advantages through multi-target and multi-pathway interactions. Traditional Chinese Medicine (TCM) formulations are inherently synergistic, yet systematically identifying their active anti-aging combinations remains a major challenge. Here, we developed DeepSMCA, a deep learning-based framework integrating molecular descriptors, ADMET parameters, and protein-protein interaction (PPI) network embeddings learned via a variational graph auto-encoder (VGAE), combined with a ResNetDNN classifier and the Bliss independence model, to identify synergistic combinations of anti-aging compound from the Dengzhan Shengmai (DZSM) formulation. Trained on 914 curated compounds, DeepSMCA achieved an area under the curve (AUC) of 0.9849 on the validation set, outperforming conventional machine learning and deep learning baselines, and interpretability analysis revealed that PPI network features contributed most (59.5%) to model predictions. Chemical profiling identified 30 constituents in DZSM, from which the three top-ranked synergistic combinations (Com1–3) were validated in D-galactose (D-Gal)-induced senescent PC12 cells. All three combinations enhanced cell viability, alleviated oxidative stress, attenuated intracellular reactive oxygen species accumulation, and decreased senescence-associated *β*-galactosidase-positive cells by up to 54.79%. Transcriptomic analysis showed that the combinations reversed 1,001–1,037 D-Gal-induced differentially expressed genes (DEGs), which were enriched in 18 shared aging-related pathways centered on longevity regulation, FoxO, p53, and autophagy signaling. Compound-target–aging-pathway network analysis further revealed complementary target engagement among constituents. This study establishes an interpretable, proof-of-concept computational–experimental pipeline for dissecting multi-component synergy in complex formulations, providing a generalizable strategy for anti-aging drug discovery from TCM.

## Introduction

Aging is a complex, irreversible process characterized by the gradual decline of physiological functions in all living organisms, leading to an increased susceptibility to chronic diseases and geriatric syndromes [1,2]. Despite significant progress, drug discovery for anti-aging therapies still faces numerous challenges, largely due to the complexity of the aging process and the unclear mechanisms underlying it [3,4]. The intricate nature of aging and the uncertainties surrounding its pathological mechanisms make traditional therapies targeting single pathways insufficient for addressing geriatric syndromes, which involve the interplay of multiple systemic dysfunctions [5]. Among various anti-aging strategies, the use of synergistic compound combinations offers distinct advantages by overcoming the limitations of single components through multi-target and multi-pathway interactions [6]. For instance, one of the earliest senolytic combinations, dasatinib plus quercetin (D + Q), has shown in preclinical models to delay, prevent, alleviate, or treat multiple senescence-associated disorders [7]. This synergistic approach not only improves individual component efficacy but also minimizes potential risks by aligning with the complex regulatory networks of aging [8]. Given the current state of anti-aging research, establishing systematic frameworks for identifying novel therapeutic combinations involving synthetic chemicals, biological molecules, and natural products is critical for advancing the field and addressing the complex challenges of aging-related diseases.

Traditional experimental methods for evaluating synergistic compound combinations are often resource- and time-intensive, creating significant bottlenecks in drug discovery and development [9]. Conventional techniques, such as checkerboard assays [10], broth microdilution [11], and dose-response matrix screenings [12], require laborious, iterative testing of various concentration ratios and extended incubation periods. Fortunately, with the rapid advancement of computing technologies, artificial intelligence (AI) strategies, including machine learning (ML) and deep learning (DL) algorithms [13], have quickly evolved to mimic human-like learning and reasoning processes, enabling the capture of knowledge from multi-scale data in drug discovery and screening [14]. Among these techniques, DL-based approaches for predicting synergistic compound combinations have suggested exceptional ability in extracting critical features and recognizing complex patterns from large-scale biomedical data, significantly outperforming traditional models [15]. DL frameworks such as DeepMDS leverage nonlinear hierarchical representations to capture multi-compound interactions directly from high-dimensional data. Additionally, Shinsuke Yuasa’s team proposed a Deep Learning-Based Senescence Scoring System by Morphology (Deep-SeSMo), a morphology-based convolutional neural network (CNN) system for predicting anti-senescent reagents. Nonetheless, most predictive models focus only on single-scale features (e.g., molecular structure) of compounds, overlooking the overall therapeutic properties of the compounds, which can limit predictive performance.

Traditional Chinese Medicine (TCM) formulations, inherently designed as synergistic combinations of multiple herbal components, offer significant advantages in both theoretical frameworks and practical applications for health preservation, care, and anti-aging. Dengzhan Shengmai (DZSM), a formulation derived from “Shengmai San”, primarily consists of four TCM ingredients: the medicinal part of *Erigeron breviscapus (Vaniot) Hand.-Mazz. (rhizoma)*, the medicinal part of *Panax ginseng C.A. Mey., (rhizoma)*, the medicinal part of *Ophiopogon japonicas (Thunb.) Ker-Gawl.(tubers)*, and the medicinal part of *Schisandra chinensis (Turcz.) Baill., (dried fruit)*, in a weight ratio of 5:1:1:1.8 (drug approval number in the Chinese Food and Drug Administration: Z20026439). Combined with expansion imaging analysis and ML algorithms, their study revealed that DZSM attenuates aging by enhancing the microstructural integrity of gray/white matter and optimizing brain network topology in elderly individuals [16]. Our previous work also confirmed that DZSM significantly enhances spontaneous activity, exploratory behavior, and learning-memory capacity in D-galactose (D-Gal)-induced subacute aging model mice. It notably reduces the increase in apoptotic-like cells and the shrinkage of Nissl bodies in the hippocampus, while rectifying the expression of differentially expressed genes (DEGs) in brain tissue as revealed by transcriptomic-network analysis [17]. Despite consensus on the pharmacodynamic effects of DZSM, comprehensive screening of synergistic compounds with anti-aging pharmacological activities and further elucidation of their material base remain significant challenges.

**Fig 1:**
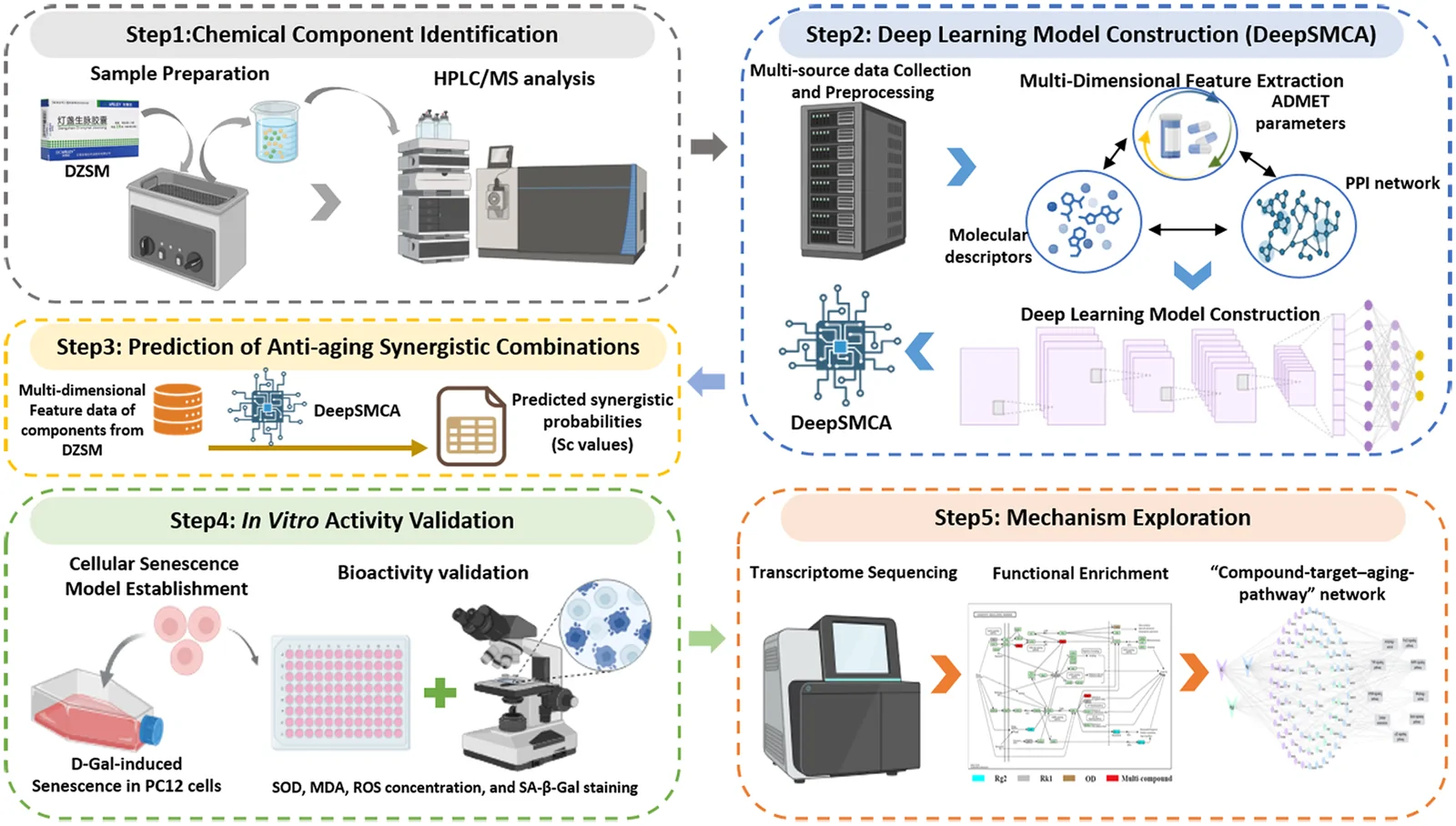
Schematic overview of the integrated workflow for AI-driven identification and experimental validation of synergistic anti-aging compound combinations from the Dengzhan Shengmai (DZSM) formulation.

This study developed a DL-based model, DeepSMCA, for predicting synergistic multi-compound combinations for anti-aging. This model integrates multi-scale features, including molecular structure, metabolic processes (ADMET parameters: absorption, distribution, metabolism, excretion, and toxicity), and protein-protein interaction (PPI) networks to identify synergistic combinations targeting anti-aging. More importantly, the identified superior combinations were validated through *in vitro* cellular assays, and their synergistic mechanisms were further explored via RNA-seq analysis and network analysis. In conclusion, this study provides a novel strategy for effectively identifying superior synergistic combinations of anti-aging compounds in the DZSM formulation and understanding their complex interactions within TCM (Fig 1).

## Materials and methods

### Chemicals and reagents

DZSM formulation dry powder voucher specimens were provided by Yunnan Bio Valley (China, No: 20190023). D-Gal (purity ≥ 99%) and DMSO was purchased from Sigma-Aldrich (St. Louis, United States), melatonin (purity ≥ 99%), and rapamycin was sourced from MedChemExpress Co., Ltd (Shanghai, China). Ginsenoside Rg1 (Rg1), Ginsenoside Rg2 (Rg2), Ginsenoside Rk1 (Rk1), Scutellarin (SC), Ophiopogonin D (OD), and Schisandrin A (SA) were obtained from Aladdin Reagents Co., Ltd. (Shanghai). PC12 cells were purchased from Pu-nuo-sai Life Technology (Wuhan, China). Dulbecco’s Modified Eagle Medium/High Glucose (DMEM) and Fetal Bovine Serum (FBS) were provided by Adamas Life (Shanghai, China). Cell Counting Kit-8 (CCK-8), malondialdehyde (MDA), superoxide dismutase (SOD), reactive oxygen species (ROS) assays, and SA-*β*-Gal staining kits were purchased from Biyuntian Biotechnology (Beijing, China).

### Sample preparation and HPLC/MS analysis

DZSM formulation dry powder (0.20 g) was ultrasonically extracted with 40 mL of 50% (v/v) methanol for 60 minutes at room temperature. The supernatant was centrifuged at 12,000 × g for 10 minutes and filtered through a 0.22 μm polytetrafluoroethylene (PTFE) membrane prior to LC-MS analysis. LC-MS analysis was performed on an Agilent 1290 Infinity II HPLC system coupled with a Q Exactive Plus Orbitrap mass spectrometer (Thermo Fisher Scientific, San Jose, CA, USA), equipped with a heated electrospray ionization (HESI-II) source operating in both positive and negative ion modes. Chromatographic separation was achieved on a Leapsil C18 column (2.7 μm, 150 mm × 2.1 mm; Thermo Fisher Scientific) maintained at 40°C. The mobile phase consisted of 0.1% formic acid in water (A) and 0.1% formic acid in acetonitrile (B). The gradient elution program was as follows: 0–1 min, 5%–15% B; 1–29 min, 15%–30% B; 29–31 min, 30%–32% B; 31–37 min, 32%–38% B; 37–48 min, 38%–43% B; 48–54 min, 43%–55% B; 54–70 min, 55%–70% B; 70–76 min, 70%–90% B; 76–86 min, 90% B. The flow rate was 300 μL/min, and the injection volume was 2 μL. For mass spectrometry, the HESI-II source parameters were optimized as follows: spray voltage, 3.5 kV in positive mode and 3.2 kV in negative mode; capillary temperature, 350°C; probe heater temperature, 400°C; sheath gas flow rate, 40 arbitrary units (arb); auxiliary gas flow rate, 10 arb; S-lens RF level, 50. Data acquisition was performed in Full MS/data-dependent MS^2^ (dd-MS^2^) mode. Full-scan mass spectra were acquired over the range of m/z 100–1,700 at a mass resolution of 70,000 full width at half maximum (FWHM, defined at m/z 200). The automatic gain control (AGC) target was set to 3 × 10^6^ with a maximum injection time (IT) of 100 ms. For dd-MS^2^, the top 5 most intense precursor ions were selected for fragmentation using higher-energy collisional dissociation (HCD) with normalized collision energies (NCE) of 20, 40, and 60 eV. MS/MS spectra were acquired at a resolution of 17,500 with an AGC target of 1 × 10^5^ and a maximum IT of 50 ms. Dynamic exclusion was set to 10.0 seconds. Raw data were processed using Compound Discoverer 3.2 (Thermo Fisher Scientific) for retention time alignment, peak detection, and deconvolution. Compound identification was performed through a multi-step strategy: (i) matching accurate mass (mass error < 5 ppm) and isotopic patterns against the mzCloud database (version 2.0, Thermo Fisher Scientific; similarity score ≥ 80%); (ii) comparison of retention times and MS/MS fragmentation patterns with authentic reference standards where available; and (iii) manual verification of elemental formulas using the built-in ChemSpider and PubChem search algorithms. A total of 30 compounds were confidently identified and are listed in Table 1 (accurate m/z values are provided to four decimal places).

**Table 1:** Results of HPLC/MS analysis of DZSM formulation.

NO.	Rt (min)	m/z	Formula	Mode	Ingredients
1	2.61	180.1413	C_9_H_8_O_4_	[M+H]^+^	Caffeic acid
2	8.40	288.2285	C_15_H_12_O_6_	[M+H]^+^	Eriodictyol
3	8.84/12.75	448.3317	C_21_H_20_O_11_	[M+H]^+^/ [M-H]^−^	Naringenin-7-glucuronide
4	9.06/13.90	462.3109	C_21_H_18_O_12_	[M+H]^+^/ [M-H]^−^	Scutellarin
5	11.69	516.4020	C_25_H_24_O_12_	[M+H]^+^	4,5-Dicaffeoylquinic acid
6	12.05/9.82	286.2128	C_15_H_10_O_6_	[M+H]^+^/ [M-H]^−^	Luteolin
7	13.13	396.6479	C_28_H_44_O	[M+H]^+^	Ergosterol
8	13.51/15.12	270.2179	C_15_H_10_O_5_	[M+H]^+^/ [M-H]^−^	Apigenin
9	33.30	800.9551	C_42_H_72_O_14_	[M+H]^+^	Ginsenoside Rg1
10	38.56	784.9602	C_42_H_72_O_13_	[M+H]^+^	Ginsenoside Rg2
11	45.65	432.4792	C_24_H_32_O_7_	[M+H]^+^	Schisandrol A
12	51.06	416.4368	C_23_H_28_O_7_	[M+H]^+^	Gomisin O
13	54.51	766.9496	C_42_H_70_O_12_	[M+H]^+^	Ginsenoside Rk1
14	54.54	514.5287	C_28_H_34_O_9_	[M+H]⁺	Gomisin F
15	56.23	400.4419	C_23_H_28_O_6_	[M+H]^+^	Schisandrin
16	66.91	416.4843	C_24_H_32_O_6_	[M+H]^+^	Schisandrin A
17	3.57	382.2771	C_17_H_18_O_10_	[M-H]^−^	4-CDOA
18	4.28	354.2712	C_16_H_18_O_9_	[M-H]^−^	4-CQA
19	7.50	678.5327	C_34_H_30_O_15_	[M-H]^−^	1,3,5-triCQA
20	10.93	446.3160	C_21_H_18_O_11_	[M-H]^−^	Breviscapine
21	22.14	947.0791	C_48_H_82_O_18_	[M-H]^−^	Ginsenoside Re
22	24.91	558.4345	C_27_H_26_O_13_	[M-H]^−^	acetyl-di-CQA
23	37.02	638.8362	C_36_H_62_O_9_	[M-H]^−^	Ginsenoside F1
24	38.01	1211.2737	C_58_H_98_O_26_	[M-H]^−^	Ginsenoside Ra1
25	38.05	1079.1764	C_53_H_90_O_22_	[M-H]^−^	Ginsenoside Rb1
26	48.68	620.8256	C_36_H_60_O_8_	[M-H]^−^	Ginsenoside Rh4
27	54.77	486.5228	C_27_H_34_O_8_	[M-H]^−^	Schisandrin F
28	56.53	854.9512	C_44_H_70_O_16_	[M-H]^−^	Ophiopogonin D
29	64.63	808.9822	C_44_H_72_O_13_	[M-H]^−^	Ginsenoside Rs4
30	65.42	808.9822	C_44_H_72_O_13_	[M-H]^−^	Ginsenoside Rs5

### DeepSMCA -based model analysis

#### Deep learning model framework.

**Fig 2:**
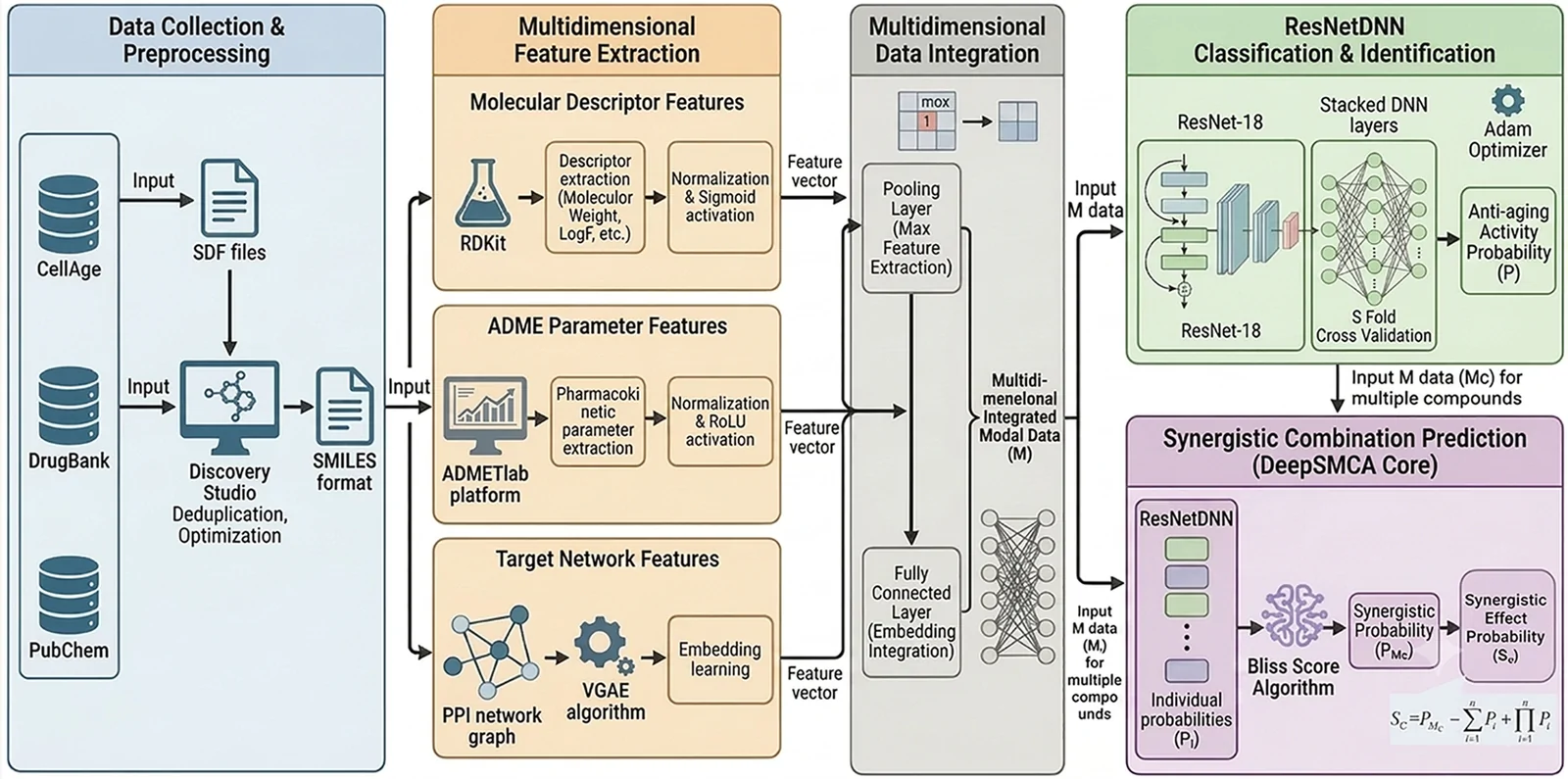
Architecture of the DeepSMCA framework for predicting synergistic anti-aging compound combinations.

The framework of DeepSMCA, a DL-based model for predicting anti-aging synergistic compound combinations, is illustrated in Fig 2, the architecture of DeepSMCA comprises: (i) a multi-dimensional feature integration and anti-aging compound identification module built upon a ResNetDNN classifier, and (ii) a synergistic combination prediction module employing the Bliss independence model. Essentially, DeepSMCA predicts the most potent anti-aging compounds as well as the optimal prospective synergistic combinations, providing an innovative methodology for the systematic and precise identification of anti-aging compound combinations.

#### Data collection and preprocessing.

The Anti-Aging and No-Anti-Aging compounds were retrieved from several databases, including CellAge (https://genomics.senescence.info/cells/), DrugBank (https://go.drugbank.com/), DrugAge (https://genomics.senescence.info/drugs/), and Aging Atlas (https://ngdc.cncb.ac.cn/aging). A comprehensive literature search was conducted using keywords such as “anti-aging," “anti-senescence,” “anti-senescent,” “prolong life,” “extend lifespan,” “prolong lifespan,” “promote longevity”, “accelerating aging”, and “accelerating senescence” across PubMed (https://www.ncbi.nlm.nih.gov/pubmed/), Science Online (http://www.sciencemag.org/), Elsevier ScienceDirect (https://www.sciencedirect.com), WANFANG DATA (https://www.wanfangdata.com.cn), and other publisher databases, focusing on the period from 1999 to 2025. Duplicates from different sources and compounds lacking structural data were excluded. The three-dimensional structural files (SDF format) of compounds were then retrieved from PubChem (https://pubchem.ncbi.nlm.nih.gov/), a leading global database of chemical and biological data. These SDF files, which detail atomic coordinates, bonding information, and structural attributes, are essential for molecular modeling and analysis. To ensure the accuracy and uniqueness of these structures, Discovery Studio 4.0 was utilized to optimize the SDF files using the “Prepare Ligands” function. This process included visualization, energy minimization, and removal of duplicate structures, enhancing the data’s reliability and quality for subsequent research. Among the retained compounds, 698 were labeled as “Anti-Aging” (positive) based on experimental evidence from the DrugAge database and published literature demonstrating lifespan extension or senescence inhibition, while 216 were labeled as “No-Anti-Aging” (negative) based on the absence of such evidence, yielding a positive-to-negative ratio of approximately 3.23:1. To address this class imbalance, a class-weighted cross-entropy loss function was employed during model training, with weights inversely proportional to class frequency. The dataset was divided into training and validation sets at an 80:20 ratio using stratified sampling to preserve the class distribution in both subsets. A five-fold stratified cross-validation strategy was adopted to ensure robust and unbiased evaluation of model performance.

#### Construction of a multidimensional effect integration data.

**Acquisition of molecular descriptor feature data:** The compounds were introduced into the PubChem database to retrieve their corresponding Simplified Molecular Input Line Entry System (SMILES) representations, which provide a concise molecular description suitable for computational processing and analysis. RDKit was then employed to generate various molecular descriptors, including molecular weight, topological polar surface area, LogP value, hydrogen bond acceptors and donors, and rotatable bonds. These descriptors serve as quantitative indicators essential for compound screening, efficacy prediction, and cheminformatics research (S1 Table). To standardize these data, the Max-Min normalization method was applied, as illustrated in formula (1), where X represents the original sample value, Min is the minimum value, Max is the maximum value, and X’ is the normalized result. Additionally, to improve the nonlinear representation of the data, the Sigmoid activation function was applied to process the standardized data, mapping it to the [0,1] interval and introducing nonlinearity to facilitate the model’s capture of complex feature relationships. The processed two-dimensional feature matrix data thus provides a solid foundation for subsequent model training and predictive analysis, aiding in the screening and optimization of compounds.


$$\begin{array}{c} X^{\prime }=\frac{X-Min}{Max-Min} \end{array}$$ (1)

**Acquisition of ADMET parameter feature data:** After obtaining the SMILES format, compounds were uploaded to ADMETlab (https://admetmesh.scbdd.com/), an integrated virtual screening platform designed to predict key pharmacokinetic parameters of compounds, including absorption (A), distribution (D), metabolism (M), excretion (E), and toxicity (T). The platform provided detailed insights into compound behavior, such as oral absorption rates, bioavailability, blood-brain barrier permeability, hepatic metabolism rates, and renal excretion half-lives (S2 Table). To standardize the diverse scales of ADMET parameters, Max-Min normalization was applied, followed by processing with the ReLU activation function, which retains positive values and sets negative ones to zero, introducing sparsity and non-linearity. This approach enhances model training efficiency and improves the model’s ability to capture complex relationships. The resulting two-dimensional feature matrix, containing pharmacokinetic parameters, serves as input for subsequent DL-based predictions and optimization of components, ensuring data consistency, model generalization, and predictive accuracy, thereby providing a solid foundation for future drug discovery and optimization.

**Acquisition of PPI network parameter feature data based on network embedding learning:** A PPI network was constructed to describe the target interaction profile of the compound. Briefly, compound targets were collected from public databases, including HIT (http://www.badd-cao.net:2345/), ETCM (http://www.tcmip.cn/ETCM/), and SwissTargetPrediction (http://www.swisstargetprediction.ch/), and subsequently imported into the STRING database (https://cn.string-db.org/, V 10.5) to construct the PPI network, with nodes representing the targets and edges indicating the interactions between them. Based on the assumption that the similarity of interactions between nodes correlates with the similarity of the nodes themselves, the Variational Graph Auto-Encoder (VGAE) algorithm was applied to learn the deep features of the nodes and interaction edges within the network. Specifically, the network for a given compound is defined as G = (A, X), where A represents the adjacency matrix and X denotes the target characteristic matrix, which includes node information and the combined interaction score within the network. The inference model is defined as follows:


$$\begin{array}{c} q(Z|X,A)=\prod_{i=1}^{N}N(Z_{i}\text{|}\mu_{i},diag\left(\sigma_{i}^{\text{2}}\right)) \end{array}$$ (2)

$$\begin{array}{c} p(A|Z)=\prod_{i=1}^{N}\prod_{j=1}^{N}p\left(A_{ij}\text{|}Z_{i},Z_{j}\right) \end{array}$$ (3)

$$\begin{array}{c} \mathrm{\backslash stackrel}\wedge A=\sigma \left(ZZ^{T}\right) \end{array}$$ (4)

where *Z* is a latent variable, *N* is the number of targets, σ is the variance of node vector, *μ* is the vector mean of nodes, *A*_*ij*_ is the one element of A, and *Â* is the reconstructed A; The loss function of VGAE is as follows:


$$\begin{array}{c} L=E_{q(Z|X,A)}\left[logp\left(A|Z\right)-KL\left[q\left(Z|X,A\right)∥p\left(Z\right)\right]\right] \end{array}$$ (5)

where KL[q||p] is the Kullback-Leibler divergence between distribution *q* and distribution *p*.

#### Construction of an integrated model for multidimensional data.

The construction of the integrated model for multidimensional data of compounds follow these steps: First, data from the target network parameter feature, ADMET parameter feature, and molecular descriptor feature of compounds were processed through a pooling layer to extract the maximum feature vectors. Next, a fully connected layer was applied to perform “embedded” associative integration, resulting in a multidimensional integration model, referred to as M, which served as the input for the next stage.

#### Key parameter optimization and evaluation.

For the ResNetDNN model, the Adam optimization algorithm was used, with the loss function set to cross-entropy loss. A five-fold cross-validation strategy was implemented to optimize key model parameters. The parameter search ranges were defined as follows: epoch number [10, 50, 100, 150], learning rate [0.001, 0.00001], decay rate [0.001, 0.0001], and number of DNN layers [1,3,5]. This parameter optimization process was designed to enhance the model’s ability to accurately identify pharmacodynamic components based on multidimensionally integrated data, aligning with the core objective of the ResNetDNN classification and identification model established earlier. The use of five-fold cross-validation ensured robustness in parameter selection, while the clearly defined search ranges provided a reproducible foundation for subsequent experimental validation in DL-based research.

#### Construction of DeepSMCA model for prediction of synergistic combinations of anti-aging compounds.

To identify synergistic combinations of anti-aging compounds, the DeepSMCA model was developed to implement a three-stage inference pipeline. **Stage I:** The multidimensionally integrated data of each individual compound (denoted as *M*_*i*_, where N ≥ 2) for N compounds were obtained from the integrated model for multidimensional data. These *M*_*i*_ data were then individually imported into the ResNetDNN classifer to obtain the individual anti-senescence probability (denoted as *P*_*i*_). **Stage II:** The feature vectors of N combined compounds are mean-pooled to generate multidimensionally integrated model data of the N compounds (denoted as *M*_*c*_, where N ≥ 2), which is then input into the same ResNetDNN to yield the combination-level probability (denoted as *P*_*Mc*_)- reflecting the predicted efficacy of the combination acting as a unified pharmacological entity. **Stage III:** The Bliss independence model quantifies the deviation from additivity via the formula (6–7). Here, *S*_*C*_ indicates synergism (the combination outperforms the sum of individual effects). The Bliss model was selected because it assumes mechanistic independence among agents, which is biologically appropriate for multi-target natural product formulations where distinct compounds modulate complementary nodes within the cellular senescence network.


$$\begin{array}{c} M_{c}=\frac{\text{1}}{n}\sum_{i=1}^{n}M_{i} \end{array}$$ (6)

$$\begin{array}{c} S_{C}\text{=}P_{M_{C}}-\sum_{i=1}^{n}P_{i}+\prod_{i=1}^{n}P_{i} \end{array}$$ (7)

Where *P*_*i*_ denotes the anti-aging probability of the i-th individual compound output by ResNetDNN, *P*_*Mc*_ denotes the probability of the pooled combination features, *M*_*c*_ is multidimensional effect integration model data of synergistic combinations, and *S*_*C*_ denotes the final synergy probability score.

#### Model comparison.

To comprehensively evaluate the performance of the proposed DeepSMCA model, a series of baseline methods were implemented for comparative analysis. These included traditional machine learning models-Support Vector Machine (SVM, RBF kernel with balanced class weights), Random Forest (RF, 100 estimators, max depth = 10), and K-Nearest Neighbors (KNN, k = 5)- as well as a deep learning baseline, DeepSynergy [18], implemented as a three-layer fully connected network (512-256-128) with batch normalization and dropout. For fair comparison, all baseline methods received the same concatenated feature vector comprising molecular descriptors (D1), ADMET parameters (D2), and mean-pooled PPI network embeddings (D3). All methods were evaluated using five-fold stratified cross-validation on the combined dataset to ensure robust performance estimation. Additionally, six evaluation metrics, including Accuracy (ACC), Precision (PRE), Recall (REC), and F1 score (F1), AUC, and Matthews Correlation Coefficient (MCC), were employed to comprehensively assess the model’s classification performance.


$$\begin{array}{c} ACC\text{=}\frac{TP+TN}{TP+TN+FP+FN} \end{array}$$ (8)

$$\begin{array}{c} PRE=\frac{TP}{TP+FP} \end{array}$$ (9)

$$\begin{array}{c} REC=\frac{TP}{TP+FN} \end{array}$$ (10)

$$\begin{array}{c} F1=\frac{2TP}{2TP+FP\text{+}FN} \end{array}$$ (11)

$$\begin{array}{c} MCC=\frac{TP\times TN-FP\times FN}{\sqrt{\left(TP+FP)(TP+FN)(TN+FP)(TN+FN\right)}} \end{array}$$ (12)

### Model interpretability analysis

To elucidate the contribution of different feature dimensions to the model’s predictions, we performed a post-hoc interpretability analysis using SHAP (SHapley Additive exPlanations) with the KernelExplainer. A representative background set of 50 samples was selected from the training data via k-means clustering, and SHAP values were computed for 100 validation samples across the full dimensional input space (D1 + D2 + D3).

### External validation on an independent dataset

To assess generalizability beyond the training distribution, an external validation set was constructed from Geroprotectors.org (positive candidates with documented anti-aging evidence) and DrugCentral (negative controls; approved drugs No-anti-aging annotation, randomly sampled with a fixed seed). To eliminate train/test leakage, deduplication was performed at both the compound-name level and the structure level using RDKit canonical SMILES against the full training and validation sets; 12 structurally duplicated compounds carried under aliases were identified and removed. The final external set comprised 85 compounds (35 Anti-Aging compounds, 50 No-Anti-Aging compounds). Because experimentally annotated protein targets required for the PPI modality (D3) were available for only 16 external compounds, external evaluation was performed using the dual-modality (D1 + D2) submodel, for which features are fully computable for any new compound. ACC, F1, and AUC were used to evaluate classification performance.

### Prediction of synergistic combinations of anti-aging compounds from DZSM formulation

Compounds of the DZSM formulation were selected to construct the multidimensionally integrated model data. The DeepSMCA model was then employed to predict the synergistic combinations of anti-aging compounds in the DZSM formulation. The combination with the highest synergistic effect probability (*S*_*C*_) value was identified as the superior synergistic anti-aging combination, hereafter referred to as the “superior combination”.

### Comparison of synergy scoring models

To assess the robustness of synergy quantification, the Bliss independence model was benchmarked against two alternative null models: a Loewe-like additivity model and the highest single agent (HSA) model. As DeepSMCA outputs anti-aging probabilities rather than dose-response measurements, classical Loewe and ZIP models were not directly computable; probability-level adaptations of the two null hypotheses were therefore implemented, as illustrated in formulas (13) and (14):


$$\begin{array}{c} E-Loewe=min(1,P_{a}+P_{b}) \end{array}$$ (13)

$$\begin{array}{c} E-HSA=max\left(P_{a},P_{b}\right) \end{array}$$ (14)

with the corresponding synergy scores defined as $S=P_{Mc}-E$ in each case, where *P*_*a*_, *P*_*b*_, and *P*_*Mc*_ are as defined in formulas (6) and (7). Inter-model agreement was evaluated using Pearson and Spearman correlation coefficients and the overlap of the top-50 ranked synergistic combinations.

### Bioactivity validation

#### Cell culture and viability assay.

The six candidate compounds (Rg1, Rg2, Rk1, SC, OD, and SA) were selected from the HPLC-MS-identified DZSM constituents because they compose the top three DeepSMCA-predicted synergistic combinations (Com1–3). All compounds were dissolved in DMSO (final concentration ≤ 0.1%, v/v). For cytotoxicity evaluation, cells were treated with each compound over a concentration gradient (0–40 μM) for 24 h, and viability was measured using the CCK-8 assay. A working concentration of 10 μM, which was non-cytotoxic and produced the greatest proliferative effect was used in all subsequent assays, and constituents within each combination were applied at equimolar ratios. Equimolar dosing was deliberately adopted to normalize constituent stoichiometry, thereby isolating the synergistic interaction from abundance differences among constituents, consistent with common practice in proof-of-concept combination screening. Melatonin and rapamycin served as positive controls. Com1 consisted of Rg1, Rg2, and SC; Com2 consisted of Rg2, Rk1, and OD; and Com3 consisted of Rg2, OD, and SA, with the three constituents applied at a 1:1:1 molar ratio within each combination. All experiments were independently repeated at least three times.

#### Senescence model.

To induce senescence, PC12 cells were exposed to D-Gal at 20, 40, 60, or 80 mg/mL for 6, 12, 24, and 48 h, and 40 mg/mL D-Gal, which reduced cell viability by approximately 50%, was selected for subsequent experiments. For protection assays, cells were pre-treated with the individual compounds or combinations (10 μM) for 24 h before D-Gal induction.

#### SOD, MDA, and cellular ROS concentration measurement.

The content of MDA and SOD was determined using commercial assay kits following the manufacturer’s instructions. Cellular ROS concentrations were measured using ROS Assay Kits. Cells were seeded in 6-well plates (1 × 10^6^ cells/well) and treated with 10 μM of the candidate anti-aging compounds and superior combinations for 24 hours. D-Gal was then used to induce cellular senescence for an additional 24 hours. Cellular ROS levels were detected by DCFH-DA dye staining and analyzed using a CytoFlex flow cytometer.

#### SA-*β*-Gal staining.

PC12 cells (5 × 10^4^ cells/well) were seeded on 24-well plates and cultured as described above. The activity of *β*-galactosidase was determined using the SA-*β*-Gal staining kit, following the manufacturer’s protocol. Briefly, cells were fixed with staining fixative for 15 minutes, washed three times with PBS, and incubated with the SA-*β*-Gal staining working solution for 24 hours at 37°C without CO_2_. Cells were then observed under a light microscope, and the positive rate of senescent cells was calculated using ImageJ software.

### Preliminary analysis on the anti-aging synergistic mechanism of superior combinations based on transcriptomics and network analysis

#### Transcriptomic analysis.

Total RNA was extracted from PC12 cells (three biological replicates per group: Control, D-Gal, D-Gal + Com1, D-Gal + Com2, D-Gal + Com3; n = 3 × 5 = 15 samples) using TRIzol Reagent (Invitrogen) with on-column DNase I digestion. RNA quality was assessed using Bioanalyzer 2100 (RIN > 8.0 for all samples). Strand-specific mRNA libraries were prepared using the TruSeq Stranded mRNA kit and sequenced on Illumina NovaSeq 6000 (2 × 150 bp paired-end, > 20 million reads per sample).

#### Data preprocessing.

Raw reads were trimmed using Trimmomatic (v0.39) and aligned to the Rattus norvegicus genome (rn6) using HISAT2 (v2.1.0). Gene expression was quantified as transcripts per million (TPM) using StringTie (v2.1.4), followed by TMM normalization (edgeR package) to account for library size differences. Principal component analysis (PCA) and normalized expression distributions was used to verify the reliability of sequencing results.

#### Differential expression analysis and Functional enrichment analysis.

DEGs were identified using “limma” package in R. Statistical significance was defined as |log_2_FoldChange| > 1 and adjusted p-value < 0.05 (Benjamini-Hochberg FDR correction). Then, identified DEGs was uploaded to the DAVID database (https://david.ncifcrf.gov/) for functional enrichment analysis.

#### Network analysis.

To explore the underlying mechanisms of the synergistic anti-aging effects of the superior combinations, compound targets were retrieved from the Similarity Ensemble Approach Database (SEA, http://sea.bkslab.org), STITCH (http://stitch.embl.de), the Comparative Toxicogenomics Database (CTD, https://ctdbase.org/), and SwissTargetPrediction (https://www.swisstargetprediction.ch/). KEGG pathway enrichment analysis was performed on the targets of the superior combinations using Metascape (https://metascape.org/), and the synergistic relationships among key aging-related pathways were examined, particularly those related to the “Longevity regulating pathway.” The regulatory relationships between targets and pathways indicated in the KEGG pathway map were used to construct the “compound-target–aging-pathway” network, which was visualized using Cytoscape 3.7.2 software.

### Statistical analysis

Comparisons between groups were made using one-way ANOVA. Statistical significance was defined as ^*^p < 0.05 and ^**^p < 0.01 versus the Control group, and ^#^p < 0.05 and ^##^p < 0.01 versus the D-Gal group.

## Results

### HPLC/MS analysis of DZSM formulation

**Fig 3:**
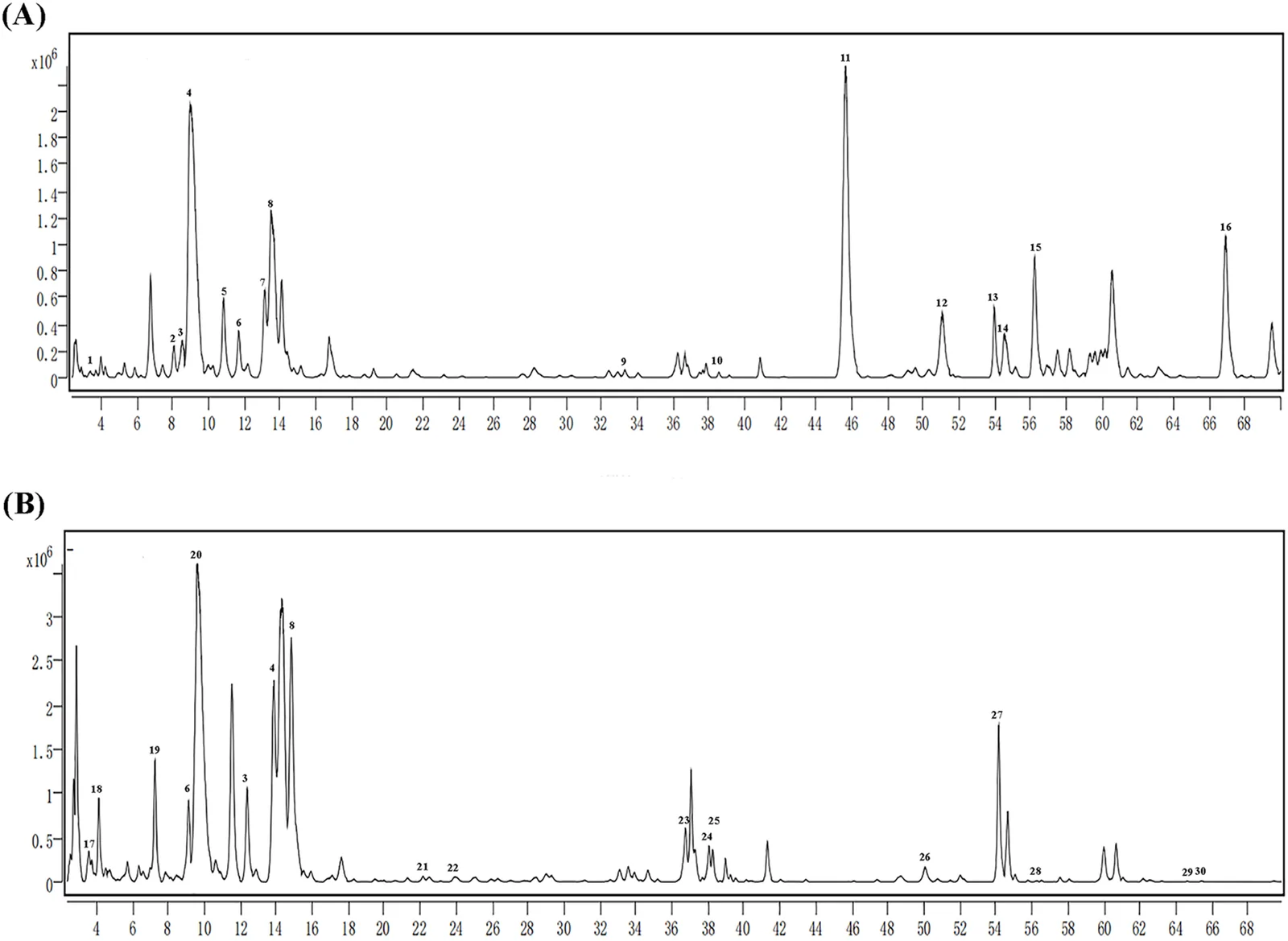
Total ion chromatograms (TICs) of the DZSM formulation acquired by HPLC–Q Exactive Plus Orbitrap MS. (A) Positive ion mode; (B) Negative ion mode.

A comprehensive analysis of the chemical compounds in the DZSM formulation extract was conducted using HPLC/MS. Through this analytical method, 30 compounds were preliminarily identified by comparing their profiles through the multi-step identification strategy described in the Methods. These compounds included Caffeic acid, Eriodictyol, Naringenin-7-glucuronide, Scutellarin, 4,5-Dicaffeoylquinic acid, Luteolin, Ergosterol, Apigenin, Ginsenoside Rg1, Ginsenoside Rg2, Schisandrol A, Gomisin O, Ginsenoside Rk1, Gomisin F, Schisandrin, Schisandrin A, 4-Caffeoyldihydrocaffeic acid, 4-Caffeoylquinic acid, 1,3,5-Tricaffeoylquinic acid, Breviscapine, Ginsenoside Re, Acetyldicaffeoylquinic acid, Ginsenoside F1, Ginsenoside Ra1, Ginsenoside Rb1, Ginsenoside Rh4, Schisandrin F, Ophiopogonin D, Ginsenoside Rs4, and Ginsenoside Rs5 (Table 1 and Fig 3). With the exception of Schisandrin A (Rt 66.91 min), no additional major peaks (signal-to-noise ratio ≥ 3) were detected in the retention time window of 67–84 min, as the 90% organic phase at 76–86 min ensured complete elution of all major constituents prior to 67 min. Regarding the formulation proportions of the constituents composing the top-ranked combination Com1 (Rg1, Rg2, and SC), SC is the principal flavonoid of *E. breviscapus*, which constitutes the largest mass fraction of DZSM (mass ratio of *E. breviscapus*, *P. ginseng*, *O. japonicus*, and *S. chinensis* = 5:1:1:1.8), whereas Rg1 and Rg2 are ginsenosides derived from *P. ginseng*. According to the Chinese Pharmacopoeia quality standard, each DZSM capsule (0.18 g) contains no less than 15.0 mg of SC as the primary quality-control marker. Validated UHPLC–MS/MS quantification of ten commercial batches further identified SC as the most abundant constituent (84.80–103.47 mg/g), followed by Rg1 (0.139–0.209 mg/g), with Rg2 present as a minor ginsenoside at lower abundance [19].

### Deep learning model analysis

#### Construction of a multidimensional integration model.

A total of 1,734 compounds were retrieved from the DrugAge, Aging Atlas, DrugBank databases, and relevant literature. The Prepare Ligands function in Discovery Studio software was used to visualize these compounds and eliminate duplicate structures, excluding those with molecular weights exceeding 1,500 Da. After filtering for compounds with complete three-dimensional feature data (molecular descriptors, ADMET parameters, and PPI network targets), 914 compounds were retained for model training and evaluation, comprising 698 Anti-Aging and 216 No-Anti-Aging compounds.

For multidimensional feature extraction, the 914 bioactive compounds were processed using the RDKit algorithm, generating 208 molecular descriptors for each compound to capture chemical structures, physical properties, and pharmacological characteristics. ADMET feature parameters were computed via the ADMETlab platform, resulting in 88 features per compound that enable in-depth analysis of *in vivo* pharmacokinetic properties. Additionally, compound targets were obtained from the HIT, SEA, and SwissTargetPrediction databases, and PPI networks were constructed for these compounds using the STRING database. The feature matrix was generated by simulating the PPI network data via the VGAE algorithm, resulting in 1,035 target network feature entries that systematically reflect their functional characteristics. Three normalized 512-dimensional vectors are stacked to construct a tensor of shape (batch, 3, 512), which is subsequently reshaped and expanded into a pseudo-image with spatial resolution 32 × 32, yielding a tensor of shape (batch, 3, 32, 32).

#### Validation of the deep learning model and model comparison.

**Fig 4:**
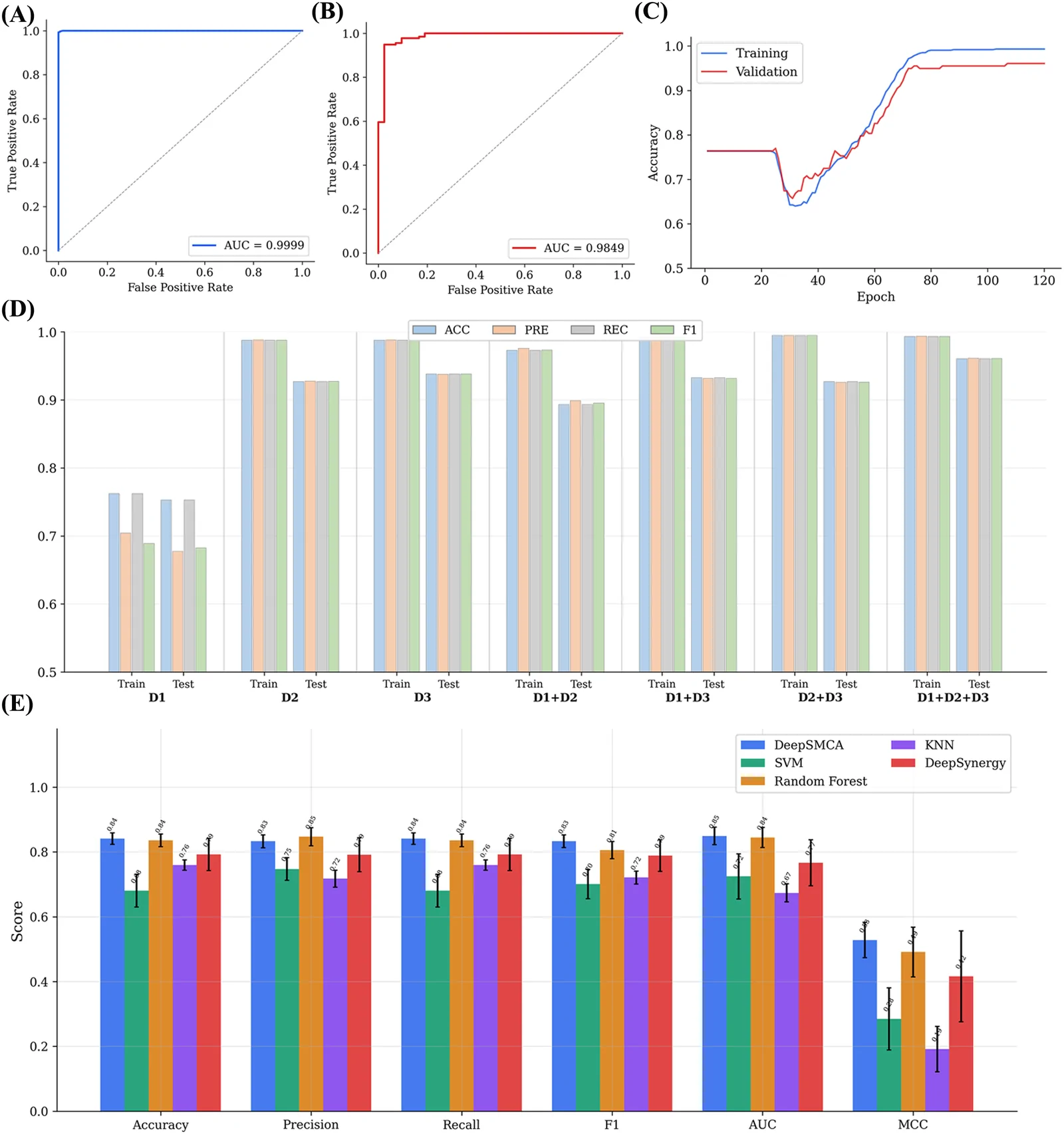
Performance evaluation and benchmarking of the DeepSMCA model. (A) Receiver operating characteristic (ROC) curve on the training set (n = 736 compounds). (B) ROC curve on the validation set (n = 178 compounds). (C) Training and validation accuracy trajectories over 120 epochs. (D) Comparative performance of single-dimensional (D1, molecular descriptors; D2, ADMET parameters; D3, PPI network embeddings), dual-dimensional (D1 + D2, D1 + D3, D2 + D3), and three-dimensional (D1 + D2 + D3) feature fusion strategies, evaluated by Accuracy (ACC), Precision (PRE), Recall (REC), and F1 score on training and validation sets. (E) Benchmark comparison of DeepSMCA against baseline methods (Support Vector Machine [SVM], Random Forest, K-Nearest Neighbors [KNN], and DeepSynergy) using five-fold stratified cross-validation (total n = 914 compounds). Data are presented as mean ± standard deviation (SD). *p < 0.05 and **p < 0.01 versus DeepSMCA. AUC, area under the curve; MCC, Matthews Correlation Coefficient.

In this study, a ResNetDNN classification model, integrating ResNet-18 with a DNN, was employed to construct a multidimensional anti-aging compound identification model. Five-fold cross-validation was utilized to optimize the key parameters of the model, and the optimal configuration was determined as follows: epoch number = 120, learning rate = 0.00001, decay rate = 0.001, and the number of DNN layers = 3 (Table 2). To further validate the generalizability and robustness of DeepSMCA, we conducted a five-fold stratified cross-validation comparison against four representative baseline methods. As shown in Fig 4(A-B), the DeepSMCA exhibited exceptional discrimination capacity, achieving AUC values of 0.9999 and 0.9849 for the training and validation sets, respectively. The training trajectory illustrated in Fig 4(C) further suggested stable convergence of classification accuracy for both datasets over 120 epochs, with the validation accuracy ultimately plateauing at approximately 0.96, indicating satisfactory generalization without substantial overfitting. In Fig 4(D), we compare predictive metrics across single-dimensional (D1, molecular descriptors; D2, ADMET parameters; D3, PPI network embeddings), dual-dimensional, and three-dimensional feature fusion strategies. While individual dimensions—particularly D2 and D3—achieved high training accuracy, their generalization to the validation set was comparatively weaker. In contrast, the full three-dimensional fusion strategy (D1 + D2 + D3) yielded the most robust and balanced performance across all evaluation metrics (ACC, PRE, REC, and F1) on both training and validation sets, underscoring the complementary value of integrating multi-scale biochemical information. Then, the performance of DeepSMCA was benchmarked against established algorithms using five-fold stratified cross-validation. As depicted in Fig 4(E), DeepSMCA attained superior performance across all six metrics compared to SVM, Random Forest, KNN, and DeepSynergy. Specifically, DeepSMCA achieved the highest AUC (0.849 ± 0.027), outperforming Random Forest (0.845 ± 0.031), DeepSynergy (0.767 ± 0.071), SVM (0.725 ± 0.070), and KNN (0.674 ± 0.028). DeepSMCA also achieved the best Accuracy (0.841 ± 0.018), F1 score (0.833 ± 0.019), and MCC (0.528 ± 0.055). Notably, DeepSMCA exhibited consistently tight error bars across all folds, whereas several baseline methods showed substantial performance variance, suggestive of overfitting to specific data partitions. These results indicated the superiority of the proposed multi-modal deep learning approach for identification of anti-aging compounds.

**Table 2:** Structure of the ResNetDNN algorithm.

NO.	ResNetDNN Algorithm
1	Input: D={(D1, S1), (D2, S2)….,(Dn, Sn)}, three dimension integrated data set of molecular feature
2	Produce molecular feature tensor
3	For i = 1 to n do
4	ResNet-18 ← load
5	ResNet-18 weights
6	ResNet-18 feature = ResNet(feature_tensor)
7	Extract the tensor features
8	End for
9	DNN model←creat_model (ResNet-18_feature)
10	DNN (F, H, A, L, δ, ω, α)
11	The model parameter for DNN
12	F: feature of each molecular compound
13	H: hidden layer (H = 3)
14	A: Activation function
15	$f_{i}(x)=maxz_{ij},z_{ij}=x^{T}W_{\mu }+b_{\mu },W\in R^{d\times m\times k}$
16	j∈[1,k]
17	L: Loss function was calculated as:
18	$L=-\frac{\text{1}}{n}\sum_{i}\left[y_{i}\cdot \text{log}\left(p_{i}\right)+\left(\text{1}-y_{i}\right)\cdot \text{log}\left(\text{1}-p_{i}\right)\right]$
19	y_i_: Real label value of the sample;
20	p_i_: Probability that the sample is predicted to be positive
21	δ: number of dimensions
22	ω: number of epochs
23	α: Learning rate
24	For all trainingSet:
25	Train test
26	End for
27	Return test p

#### Model interpretability analysis.

**Fig 5:**
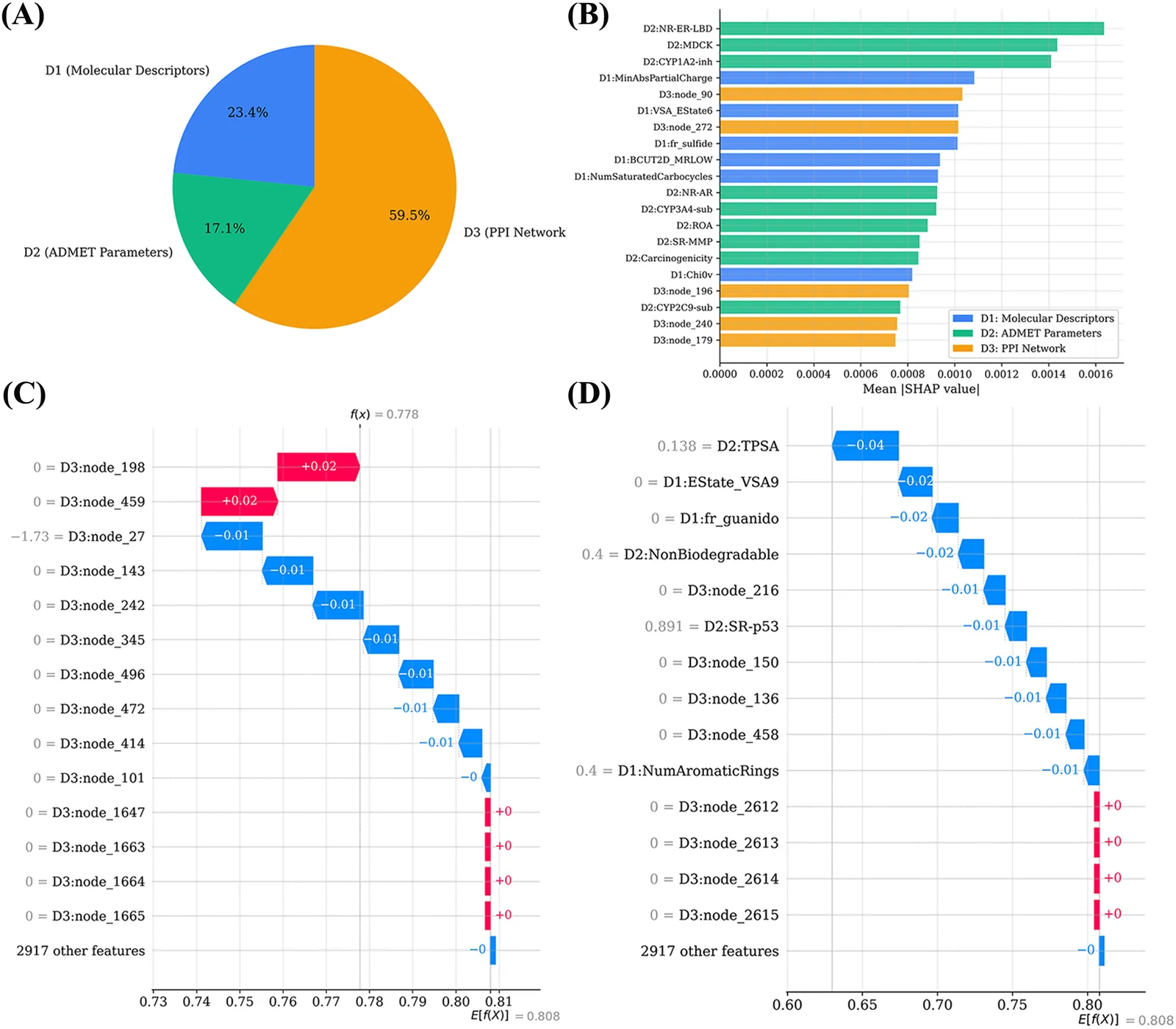
Post-hoc interpretability analysis of the DeepSMCA model using SHAP (SHapley Additive exPlanations). (A) Relative contribution of the three feature dimensions (D1, molecular descriptors; D2, ADMET parameters; D3, PPI network embeddings) to model predictions. (B) Top 20 most influential individual features ranked by mean absolute SHAP value. Representative SHAP waterfall plots illustrating feature contributions for (C) an Anti-Aging compound prediction and (D) a No-Anti-Aging compound prediction. Red and blue arrows indicate positive and negative contributions to the predicted class probability, respectively. ADMET, absorption, distribution, metabolism, excretion, and toxicity; NR-ER-LBD, nuclear receptor estrogen receptor ligand-binding domain; MDCK, Madin-Darby canine kidney cell permeability; CYP1A2-inh, cytochrome P450 1A2 inhibition; TPSA, topological polar surface area.

As shown in Fig 5(A), the aggregated SHAP analysis revealed that the PPI network dimension (D3) contributed 59.5% of the total feature importance, followed by the molecular descriptor dimension (D1) at 23.4% and the ADMET parameter dimension (D2) at 17.1%. This result provides strong empirical support for the design rationale of DeepSMCA’s multi-dimensional feature fusion architecture, indicating that network-level biological information captured through VGAE encoding plays a dominant role in identification of anti-aging compounds. The top 20 most influential individual features are presented in Fig 5(B). Among these, ADMET pharmacokinetic parameters such as NR-ER-LBD (estrogen receptor ligand-binding domain activity), MDCK (Madin-Darby canine kidney cell permeability), and CYP1A2-inh (CYP1A2 metabolic enzyme inhibition) ranked highest, indicating that pharmacokinetic properties are critical determinants for individual compound predictions. Molecular descriptors including MinAbsPartialCharge and VSA_EState6 also appeared in the top features, reflecting the importance of molecular structural characteristics. Several PPI network nodes contributed substantially, demonstrating that protein-protein interaction topology provides complementary information beyond molecular-level features. Representative SHAP waterfall plots for individual predictions are shown in Fig 5(C-D). For a correctly classified Anti-Aging compound, PPI network nodes were the primary positive contributors, while for a correctly identified No-Anti-Aging compound, ADMET features such as TPSA (topological polar surface area) drove the negative prediction, suggesting that unfavorable pharmacokinetic profiles serve as discriminative markers against anti-aging activity.

#### External validation.

On the independent external validation set (n = 85, 35 Anti-Aging/50 No-Anti-Aging), the dual modality (D1 + D2) achieved an AUC of 0.626, ACC of 0.494, F1 of 0.583. The D1 + D2 submodel was first verified on its internal validation set (AUC = 0.844) before external evaluation. The performance gap relative to internal cross-validation is attributable to the unavailability of the PPI modality (approximately 59.5% SHAP contribution) for most external compounds, domain shift between data sources-supported by a class-weighting control experiment that raised internal AUC to approximately 0.95 while external AUC remained approximately 0.63-and label false negatives among negative controls (e.g., romidepsin and baricitinib, which have reported geroprotective activity), which depress the measured specificity and AUC.

#### Prediction of synergistic combinations of anti-aging compound in DZSM formulation.

The multidimensional integrated data of bioactive compounds in the DZSM formulation were obtained by combining target network parameter features, ADMET parameter features, and molecular descriptor features. The synergistic combinations of anti-aging compound in the DZSM formulation were then predicted using the constructed DeepSMCA model. As shown in S3 Table, the predicted synergistic probabilities (*S*_*C*_ values) of the top-ranking combinations ranged from 98.18% to 99.99%, indicating robust discriminative capacity of the model in prioritizing candidate combinations. Based on prior reports, the top three synergistic combinations were selected for further analysis.

#### Robustness of synergy quantification across alternative scoring models.

To evaluate the robustness of synergy quantification to the choice of null model, Bliss-derived synergy scores were compared with Loewe-like an HSA score. Bliss scores were strongly correlated with both Loewe-like scores (Pearson r = 0.948, Spearman ρ = 0.935) and HSA scores (Pearson r = 0.955, Spearman ρ = 0.944) (Table 3). Critically, the top-50 synergistic combinations prioritized by the Bliss model overlapped by 98% with those prioritized by the Loewe-like model and by 80% with those prioritized by HSA (Table 3), demonstrating that the highest-ranked candidate combinations-the primary output of DeepSMCA-are essentially insensitive to the choice of synergy metric. The comparatively lower overlap with HSA is consistent with the deliberately conservative nature of the HSA null hypothesis, which credits a combination only for effects exceeding its most potent single constituent. These results indicate that the use of the Bliss independence model does not introduce systematic misidentification or omission of strongly synergistic combinations in this study.

**Table 3:** Concordance between the Bliss independence model and alternative synergy scoring models.

Model Comparison	Pearson	Spearman	Top-50 overlap (%)
Bliss vs. Loewe-like	0.948	0.935	98
Bliss vs. HAS	0.955	0.944	80

### Bioactivity validation

#### Candidate synergistic combinations Attenuate D-Gal-induced Senescence in PC12 cells by enhancing viability and alleviating oxidative stress.

**Fig 6:**
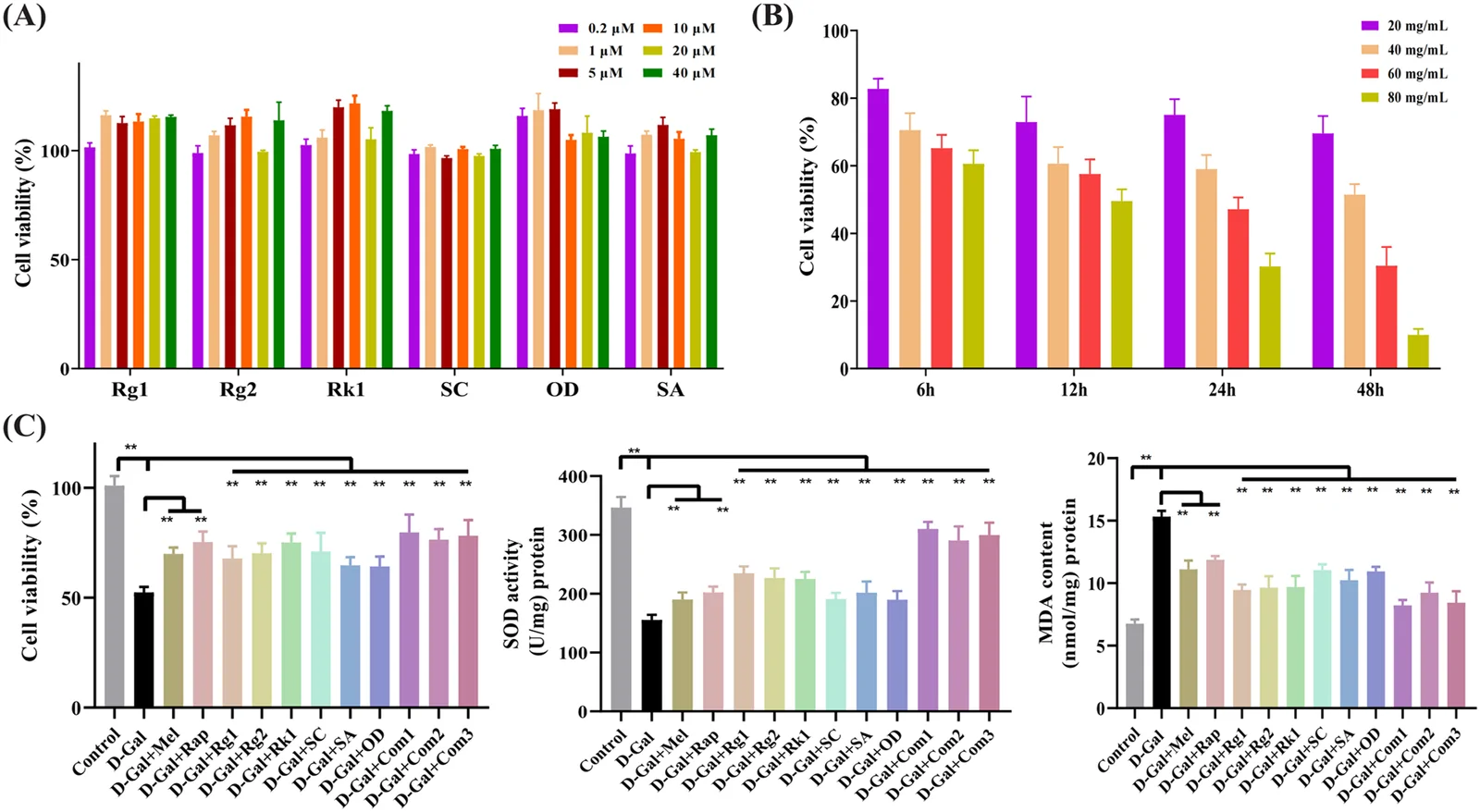
Effects of candidate compounds on D-Gal-induced PC12 cell senescence. (A) Cell viability of PC12 cells treated with individual compounds. (B) Impact of D-Gal treatments on PC12 cell viability at 6, 12, 24, and 48 hours. (C) Cell viability, SOD activity and MDA levels of senescent PC12 cells treated with individual compounds and candidate synergistic combinations. The compositions of Com1–3 are defined in the Methods. Data are presented as mean ± SD. ^*^p < 0.05, ^**^p < 0.01 vs Control; ^#^p < 0.05, ^##^p < 0.01 vs D-Gal.

CCK-8 assay was employed to assess the cytotoxicity and protective effects of individual compounds and their synergistic combinations on senescent PC12 cells. As shown in Fig 6(A), no significant cytotoxicity was observed across various concentrations of individual compounds. Instead, dose-dependent proliferative effects were noted, particularly for Ginsenosides Rg1 and Rg2. Previous studies have indicated that high levels of D-Gal induce oxidative stress by generating ROS and accelerate cellular senescence. Therefore, D-Gal-induced PC12 cells were used to investigate the anti-aging effects of individual compounds and candidate synergistic combinations. As illustrated in Fig 6(B), D-Gal at concentrations of 20, 40, 60, and 80 mg/mL significantly reduced cell viability in a dose-dependent manner compared to the Control group. Notably, 40 mg/mL D-Gal reduced cell viability by 50.47% within 48 hours (p < 0.01), and this concentration was selected for subsequent experiments.

The cell viability of synergistic combinations (at equimolar ratios) was then compared to that of individual compounds under the same conditions. As shown in Fig 6(C), both individual compounds and synergistic combinations protected cells from the D-Gal-induced viability reduction, with the synergistic combinations notably enhancing cell viability compared to individual compounds.

Aging is closely associated with oxidative stress, a key driver of cellular senescence. Consequently, oxidative stress markers-MDA and SOD-were measured in the aging cell model. The D-Gal group exhibited increased MDA production and decreased SOD activity compared to the Control group (*p* < 0.01). However, treatment with individual compounds or synergistic combinations reduced MDA levels and enhanced SOD activity relative to the D-Gal group. Specifically, synergistic combination 1 reduced MDA levels and increased SOD activity. These results demonstrate that both individual compounds and synergistic combinations significantly protect PC12 cells from D-Gal-induced viability reduction and alleviate oxidative stress in the D-Gal-induced aging model.

#### Effects of candidate synergistic combinations on D-gal-induced Senescence in PC12 cells by reducing ROS levels and SA-*β*-Gal staining.

**Fig 7:**
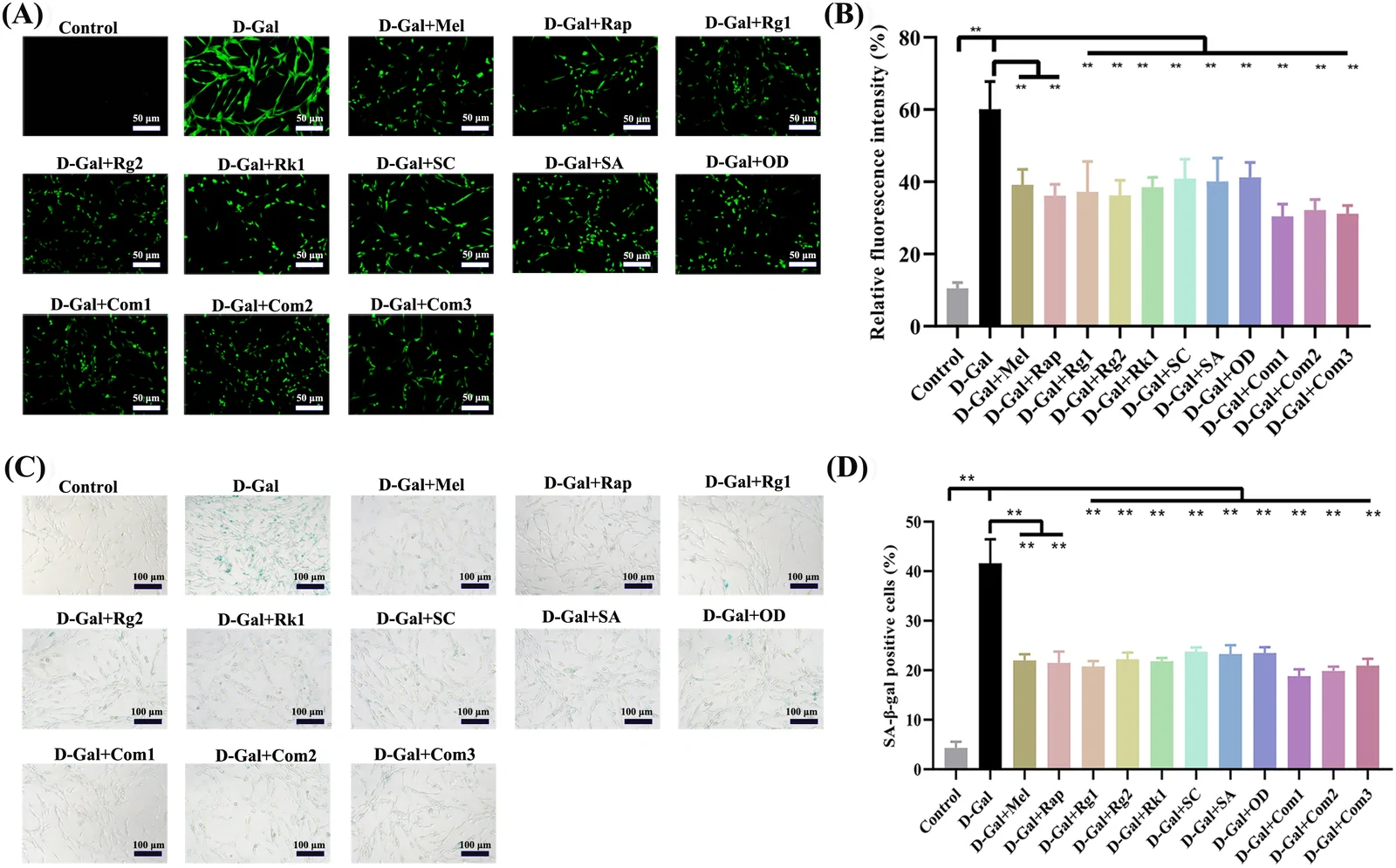
Effect of individual compounds and candidate synergistic combinations on ROS levels and cell senescence in PC12 cells. (A) Representative images of ROS levels in PC12 cells (scale bar = 50 μm). (B) Mean fluorescence intensity of ROS levels. (C) Representative images of SA-*β*-gal-stained PC12 cells (scale bar = 100 μm). (D) Proportion of SA-*β*-gal-positive cells. Data are presented as mean ± SD. Data are presented as mean ± SD. ^*^p < 0.05, ^**^p < 0.01 vs Control; ^#^p < 0.05, ^##^p < 0.01 vs D-Gal.

Given that elevated ROS levels contribute to cellular senescence, a ROS assay was conducted to evaluate the effects of synergistic combinations on ROS levels. As shown in Fig 7(A-B), the D-Gal group exhibited increased ROS levels in senescent PC12 cells compared to the Control group. Notably, pretreatment with individual compounds or synergistic combinations significantly reduced ROS levels, with synergistic combination 1 exhibiting the most pronounced effect.

Additionally, upregulation of SA-*β*-gal activity is a hallmark of cellular senescence. The results indicated that the proportion of SA-*β*-gal-positive cells was significantly lower in the individual compound- and synergistic combination-treated groups compared to the D-Gal group. Specifically, synergistic combination 1 reduced the proportion of SA-*β*-gal-positive cells by 54.79%. These results confirm that both individual compounds and synergistic combinations significantly mitigate senescence in D-Gal-treated PC12 cells (Fig 7(C-D)). Both positive controls, melatonin and rapamycin, significantly restored cell viability and SOD activity and reduced MDA levels in D-Gal-treated PC12 cells and similarly attenuated intracellular ROS accumulation and the proportion of SA-*β*-gal-positive cells, confirming the validity of the experimental system. Notably, among the three candidate combinations, Com1 consistently exhibited the greatest effects across these endpoints, with the largest increase in SOD activity, the strongest reductions in MDA levels and intracellular ROS accumulation, and the lowest proportion of SA-*β*-gal-positive cells, supporting its prioritization as the superior combination for subsequent mechanistic analyses.

### Preliminary analysis on the anti-aging synergistic mechanism of superior combinations

**Fig 8:**
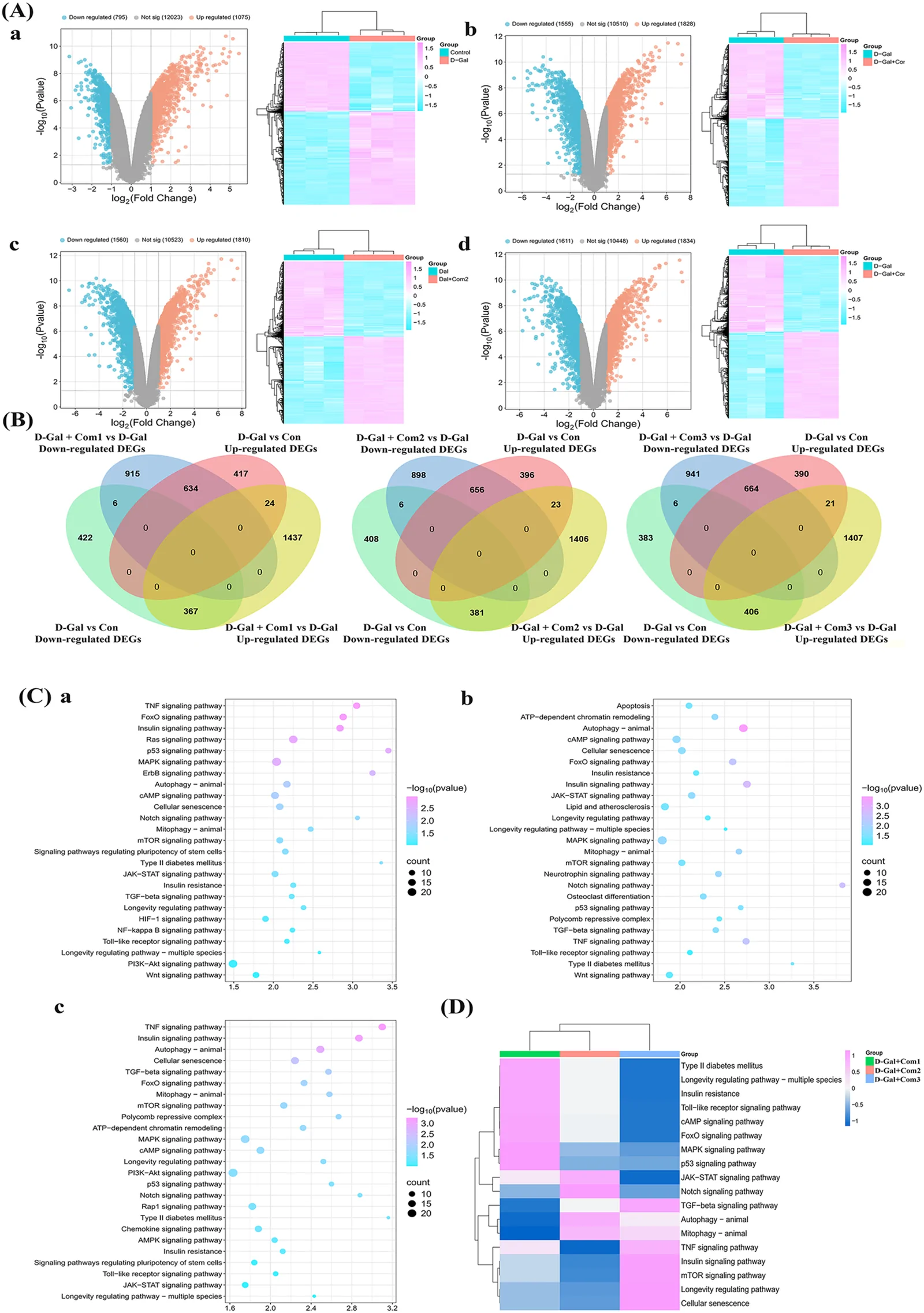
Transcriptomic analysis of changes induced by D-Gal and candidate synergistic combinations. (A) Volcano map and heatmap of DEGs: (a) D-Gal vs Control; (b) D-Gal + Com1 vs D-Gal; (c) D-Gal + Com2 vs D-Gal; (d) D-Gal + Com3 vs D-Gal. (B) Venn diagram of DEGs between D-Gal + Com1 vs D-Gal, D-Gal + Com2 vs D-Gal, and D-Gal + Com3 vs D-Gal. (C) KEGG enrichment analysis of superior combinations: D-Gal + Com1 (a), D-Gal + Com2 (b), D-Gal + Com3 (c). (D) Comparative heatmap analysis of enriched KEGG pathways for superior combinations.

As shown in Fig 8(A), a total of 1870 DEGs were identified between the D-Gal and Control groups, with 1075 upregulated and 795 downregulated genes. Following treatment with the superior combinations, 3383 DEGs were observed in the D-Gal + Com1 vs. D-Gal group (1828 upregulated and 1555 downregulated), 3370 DEGs in the D-Gal + Com2 vs. D-Gal group (1810 upregulated and 1560 downregulated), and 3445 DEGs in the D-Gal + Com3 vs. D-Gal group (1834 upregulated and 1611 downregulated). The Venn diagram in Fig 8(B) illustrates that 1001 DEGs were reversed between the D-Gal + Com1 vs. D-Gal and D-Gal vs. Control groups (634 downregulated and 367 upregulated), 1037 DEGs between the D-Gal + Com2 vs. D-Gal and D-Gal vs. Control groups (656 downregulated and 381 upregulated), and 1031 DEGs between the D-Gal + Com3 vs. D-Gal and D-Gal vs. Control groups (664 downregulated and 367 upregulated). These results suggest that the superior combinations exert anti-aging effects by reversing the expression of these DEGs.

To further explore the pathogenesis of aging and the biological changes induced by the superior combinations, functional enrichment analysis of the reversed DEGs was performed across the Control, D-Gal, D-Gal + Com1, D-Gal + Com2, and D-Gal + Com3 groups. As depicted in Fig 8(C-D), the top 25 enriched pathways among the three superior combinations revealed 18 common aging-related pathways.

These pathways can be categorized into three major groups based on their functional roles in aging regulation: (1) Core driver pathways of aging initiation and progression, which directly control “lifespan regulation” or “cellular senescence processes”. Representative pathways in this category include the Longevity regulating pathway, Longevity regulating pathway – multiple species, Cellular senescence pathway, FoxO signaling pathway, and p53 signaling pathway. (2) Essential components of the aging regulatory network, which indirectly modulate the aging process by regulating fundamental biological processes such as cellular homeostasis, stress responses, and metabolic equilibrium. Key pathways in this group include the Autophagy – animal pathway, Mitophagy – animal pathway, TNF signaling pathway, MAPK signaling pathway, mTOR signaling pathway, Insulin signaling pathway, Notch signaling pathway, TGF-beta signaling pathway, and cAMP signaling pathway. (3) Aging-associated metabolic disease pathways, which reflect the interplay between pathological states and the aging process. These pathways are key targets for investigating aging mechanisms and age-related diseases, with typical examples including Type II diabetes mellitus and Insulin resistance pathways.

**Fig 9:**
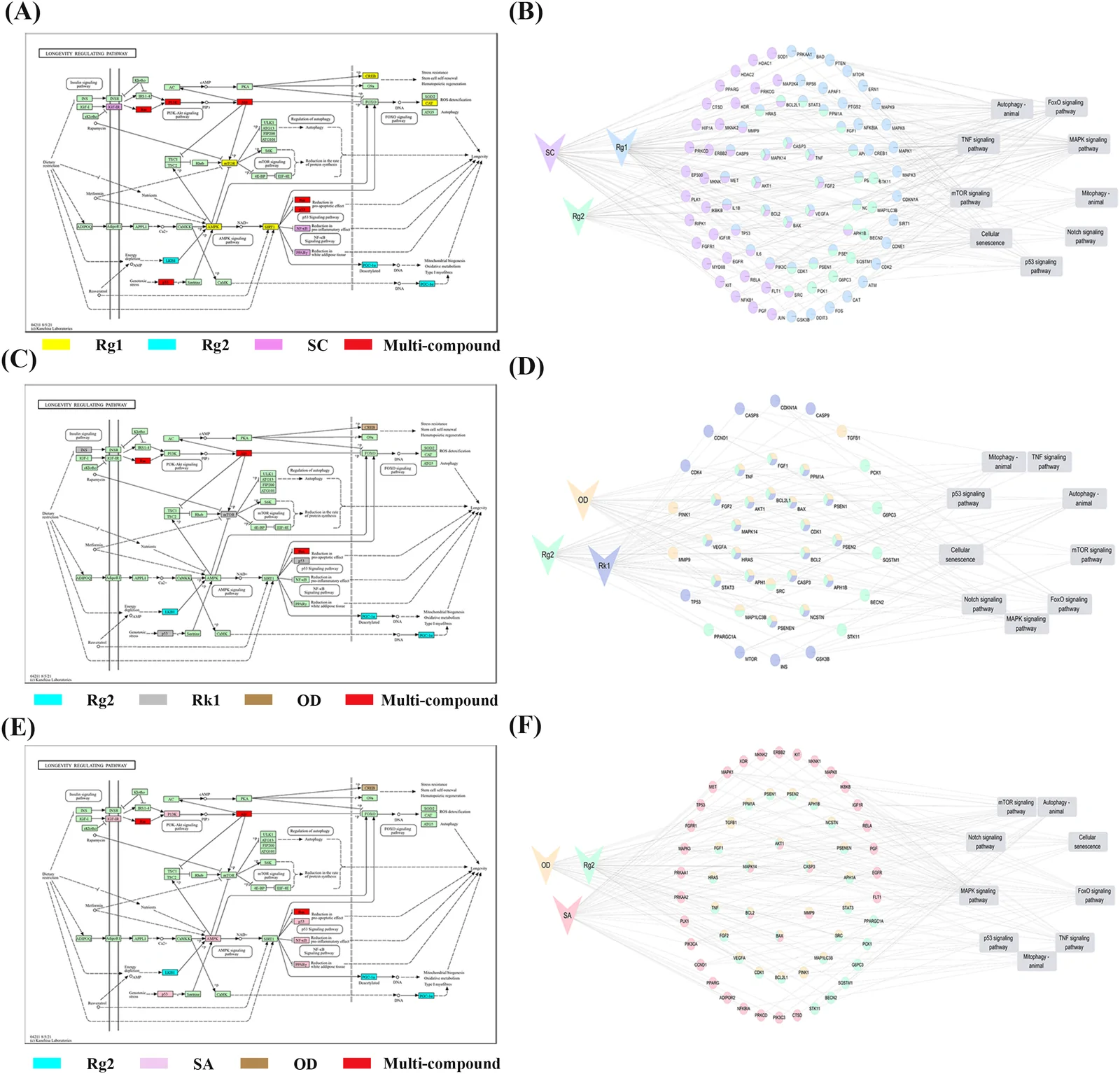
Preliminary analysis of anti-aging synergistic mechanisms of superior combinations. (A, C, E) KEGG pathway analysis of the “Longevity regulating pathway” affected by Com1 (A), Com2 (C), and Com3 (E). (B, D, F) Synergistic compound-target–aging-pathway networks for Com1 (B), Com2 (D), and Com3 (F).

Targets of superior combinations were mapped to the hsa04211 (Longevity Regulating Pathway). The enriched aging-related regulatory pathways, along with their corresponding targets and compounds, were visualized as a “compound-target–aging-pathway”network using Cytoscape software. As illustrated in Fig 9(A), within the Longevity Regulating Pathway of Com1 (Rg1, Rg2, SC), the three compounds (Rg1, Rg2, and SC) were associated with 19 targets, including 6 common targets (BAX, HRAS, TP53, PIK3CA, AKT1, and SOD1), and regulated multiple pathways such as the PI3K-Akt signaling pathway, mTOR signaling pathway, FOXO signaling pathway, AMPK signaling pathway, p53 signaling pathway, and NF-κB signaling pathway.

The “compound-target–aging-pathway” network of Com1 consisted of 99 nodes (3 compound nodes, 87 target nodes, and 9 pathway nodes) and 285 interaction edges. Rg1, Rg2, and SC synergistically regulated the MAPK, FOXO, TNF, Autophagy-animal, and p53 signaling pathways *via* shared targets, including AKT1, BAX, BCL2, CASP3, FGF2, MAPK14, TNF, and VEGFA. Rg1 exclusively targeted a broad spectrum of core components across multiple signaling pathways, with its regulatory network centered on key nodes in the MAPK, FoxO, and TNF signaling pathways (e.g., MAPK3, AKT1, RELA) as well as key regulators of cell survival and apoptosis (e.g., BCL2, CASP3, TP53). In contrast, Rg2 acted as a focused regulator of autophagic flux and mitochondrial quality control, with its functional network centered on autophagy/mitophagy-specific targets (e.g., BECN2, SQSTM1, STK11) and selective cell cycle modulators (e.g., CDK1, MAPK14). Meanwhile, SC functioned as a specialized modulator of pro-inflammatory signaling and vascular homeostasis, utilizing unique targets within the TNF signaling pathway, angiogenesis-related factors (e.g., TNF, IKBKB, VEGFA, KDR), and key growth factor receptors (e.g., EGFR, IGF1R) (Fig 9(B)).

In the Longevity Regulating Pathway of Com2 (Rg2, Rk1, OD), the three compounds were involved in 9 targets, including 3 common targets (BAX, HRAS, and AKT1), and regulated multiple pathways such as PI3K-Akt, mTOR, FOXO, AMPK, and p53, as shown in Fig 9(C). The “compound-target–aging-pathway” network of Com2 comprises 52 nodes (3 compound nodes, 40 target nodes, and 9 pathway nodes) and 156 interaction edges. The three compounds share 15 core targets and are predominantly enriched in five core signaling pathways: MAPK, Notch, FoxO, autophagy, and TNF pathways. Rk1, recognized as a “specialist in cell cycle and apoptosis regulation,” exclusively targets key components of the p53 and mTOR signaling pathways, along with critical cell cycle regulators (e.g., CASP8, CASP9, TP53, MTOR). This focus facilitates the modulation of cell proliferation, apoptosis initiation, and cellular senescence. In contrast, Rg2 serves as a regulator of autophagy and metabolic balance, with its network centered on autophagy/mitophagy-specific targets (e.g., BECN2, SQSTM1, STK11) and glucose metabolism-related factors (e.g., G6PC3, PCK1), contributing to cellular homeostasis and energy metabolism regulation. Meanwhile, OD regulates mitochondrial function and microenvironmental homeostasis, utilizing targets such as PINK1 and TGFB1 to specifically modulate mitophagy, tissue remodeling, and inflammatory responses, thus maintaining cellular microenvironmental stability (Fig 9(D)).

For Com3 (Rg2, OD, SA), the three compounds interact with a total of 14 targets, including 3 common targets (BAX, HRAS, and AKT1), and regulate multiple pathways such as PI3K-Akt, mTOR, FOXO, AMPK, p53, and NF-κB signaling pathways (Fig 9(E)). The “compound-target–aging-pathway” network of Com3 contains 71 nodes (3 compound nodes, 59 target nodes, and 9 pathway nodes) and 204 interaction edges. Rg2, OD, and SA share five core common targets (AKT1, BAX, BCL2, CASP3, MAPK14) and are significantly enriched in five core signaling pathways: MAPK, p53, TNF, Autophagy-animal, and Cellular Senescence pathways. Rg2 regulates autophagy and glucose metabolism *via* targets such as STK11, BECN2, and G6PC3. OD prioritizes mitochondrial function and microenvironmental stability through PINK1 and TGFB1. SA acts as a broad-spectrum modulator, targeting receptor tyrosine kinases (e.g., EGFR, IGF1R), PI3K pathway components (e.g., PIK3CA), and nuclear transcription factors (e.g., RELA), thereby integrating multiple signaling networks to modulate complex aging-related biological processes (Fig 9(F)). Collectively, the compounds in the superior combinations synergistically regulate multiple aging-related pathways by acting on the core biological processes of “signal transduction–cellular homeostasis–stress response.” This regulatory mode alleviates oxidative stress, inhibits cellular senescence, and restores cellular homeostasis, thereby exerting synergistic anti-aging effects.

## Discussion

The present study developed DeepSMCA, a deep learning-based framework integrating molecular descriptors, ADMET parameters, and PPI network embeddings, to systematically identify synergistic anti-aging compound combinations from the Dengzhan Shengmai (DZSM) formulation. Our findings suggested that the three top-ranked combinations (Com1-3) significantly attenuated cellular senescence phenotypes across D-Gal-induced PC12 models, as evidenced by restored redox homeostasis, reduced ROS accumulation, and diminished SA-*β*-Gal activity. Transcriptomic profiling further suggests that these combinations may exert systems-level anti-aging effects by modulating 18 core pathways centered on the “signal transduction–cellular homeostasis–stress response” axis. Collectively, this work establishes a proof-of-concept pipeline bridging computational prediction and experimental validation for dissecting multi-component synergy in complex traditional Chinese medicine formulations.

In this study, 30 bioactive compounds in the DZSM formulation were identified through HPLC-MS analysis (Table 1), including ginsenosides, scutellarin, and ophiopogonin D. This provides a foundational chemical library for subsequent synergy prediction, aligning with previous phytochemical studies on DZSM that highlighted ginsenosides and flavonoids as the primary active components [20].

A central innovation of DeepSMCA lies in its multi-dimensional feature fusion strategy. Conventional synergy prediction models, such as DeepSynergy and DrugCell, have primarily relied on single-scale chemical or genomic features, which may overlook the polypharmacological nature of anti-aging interventions. By incorporating PPI network embeddings learned through variational graph auto-encoder encoding, DeepSMCA captures target-level topological information that reflects the systems pharmacology of aging.

The choice of null model is a recognized source of variability in synergy quantification, and no single reference framework is universally considered optimal. The Bliss independence model was adopted in this study because its assumption of mechanistic independence among agents is biologically appropriate for multi-target natural product formulations, in which distinct compounds modulate complementary nodes of the cellular senescence network. Importantly, the cross-model benchmarking performed here demonstrated that synergy scores and—most critically—the ranking of the strongest synergistic combinations were highly consistent across the Bliss, Loewe-like, and HSA frameworks (Pearson ≥ 0.948; top-50 overlap ≥ 80%). This indicates that the prioritization of candidate combinations by DeepSMCA reflects an intrinsic property of the predicted combination-level probabilities rather than an artifact of the Bliss independence assumption. The modest discrepancy observed for HSA (80% top-50 overlap) is attributable to its conservative null hypothesis, which does not credit combinations whose advantage arises from complementary multi-target engagement rather than from exceeding the most potent single agent. Notably, the three constituents of Com1 originate from the two predominant drugs of the formulation: SC is the quality-control marker and most abundant constituent of DZSM, and Rg1 has been proposed as one of nine chemical markers for DZSM quality evaluation [19].

*In vitro* bioactivity validation demonstrated that the top three synergistic combinations identified by DeepSMCA significantly improved the viability of D-Gal-induced senescent PC12 cells, reduced MDA and ROS levels, and enhanced SOD activity. Among them, Com1–3 consistently reversed oxidative stress markers and senescence-associated phenotypes, with Com1 exhibiting the most robust efficacy. These observations are biologically plausible given the known pharmacological profiles of the constituent compounds. Ginsenosides Rg1 and Rg2 have been reported to attenuate oxidative stress and enhance mitochondrial function through AMPK/Nrf2 and autophagy pathways, while scutellarin exhibits potent anti-inflammatory and neuroprotective effects via modulation of the AKG-IDH1 axis and suppression of SASP. The inclusion of OD and SA, which target mitophagy and ferroptosis pathways, respectively, likely expands the mechanistic coverage of the combinations.

These synergistic combinations exerted anti-aging effects by alleviating oxidative damage, consistent with previous reports on the antioxidant properties of ginsenosides [21] and scutellarin [22]. Notably, the efficacy of the synergistic combinations was significantly higher than that of individual compounds, highlighting the advantages of multi-component synergy in anti-aging therapy. These findings support the TCM theory of “Jun-Chen-Zuo-Shi” (monarch-minister-assistant-guide), wherein multiple components work together to enhance efficacy and reduce side effects [23].

Transcriptomic and network analyses elucidated the molecular mechanisms underpinning the anti-aging effects of the synergistic combinations. As shown in Fig 8, transcriptomic profiling revealed that D-Gal treatment significantly altered the expression of 1870 genes compared to the Control group, indicating that D-Gal induces a broad transcriptomic reprogramming contributing to aging onset and progression [24]. Notably, treatment with the three superior combinations reversed the expression of numerous D-Gal-regulated genes, with 1001, 1037, and 1031 DEGs being reversed in the Com1, Com2, and Com3 treatment groups, respectively. These findings strongly suggest that the superior combinations exert their anti-aging effects by restoring the aberrant gene expression profiles induced by D-Gal, providing a molecular foundation for their activity. To further investigate the biological implications of the reversed DEGs, functional enrichment analysis identified 18 shared aging-related pathways across the three superior combination groups. These pathways were categorized into three core modules: (1) driver pathways of aging initiation and progression, (2) essential components of the aging regulatory network, and (3) aging-associated metabolic disease pathways. The Longevity regulating pathway, a central regulatory axis of aging, was particularly prominent. This pathway is evolutionarily conserved and plays a pivotal role in lifespan modulation by integrating multiple intracellular and extracellular signals [25]. The FoxO family of transcription factors, key downstream effectors of this pathway, regulates genes involved in oxidative stress resistance, DNA repair, and cell cycle arrest, thereby delaying cellular senescence [26]. The p53 signaling pathway, often referred to as the “guardian of the genome,” triggers cell cycle arrest or apoptosis in response to DNA damage, with its dysregulation being a hallmark of aging and age-related diseases [27]. Autophagy and mitophagy, vital processes for maintaining cellular homeostasis, remove damaged organelles and misfolded proteins. Their decline is a key contributor to aging [28]. The mTOR signaling pathway, a central regulator of cell growth and metabolism, tightly controls autophagy; its hyperactivation suppresses autophagy, leading to the accumulation of cellular debris and promoting aging [29]. Additionally, the TNF signaling pathway, which mediates inflammatory responses, plays a significant role in inflammaging—a chronic, low-grade inflammation associated with aging that exacerbates cellular dysfunction [30]. The Type II diabetes mellitus and insulin resistance pathways highlight the link between aging and metabolic disorders, with aging often accompanied by declining insulin sensitivity, which disrupts glucose metabolism and energy homeostasis, further accelerating aging [31]. The enrichment of these pathways among the reversed DEGs suggests that the superior combinations target key regulatory nodes of aging initiation, inhibiting cellular senescence and lifespan shortening, enhancing cellular stress resistance, and improving metabolic balance to reverse aged phenotypes. These findings support the potential of these combinations as strategies for managing aging-related metabolic diseases.

The “compound-target–aging-pathway” network was constructed to further elucidate the synergistic anti-aging mechanism of the compounds in each superior combination. The synergistic effects of Com1 (Rg1, Rg2, SC) manifest through multi-dimensional regulation, spanning from intracellular signal transduction to modulation of the tissue microenvironment. Rg1 plays a broad-spectrum regulatory role, particularly targeting key nodes within the MAPK, FoxO, and TNF signaling pathways, providing a fundamental framework for the combination to regulate cell survival, apoptosis, and inflammatory responses [32]. Notably, the activation of the PI3K-Akt signaling pathway by ginsenosides is one of the core mechanisms underlying their neuroprotective and muscle-regulatory effects [33]. Through similar mechanisms, Rg1 synergizes with Rg2 and SC to enhance Com1’s positive regulation of cell survival pathways. Previous studies have highlighted the core mechanism by which ginsenoside Rg2 regulates the autophagy-mitochondrial axis, initiating the autophagic program at both the transcriptional and signaling levels by activating ULK1 kinase [34] and upregulating TFEB1 [35]. This coordinated action facilitates the effective clearance of dysfunctional mitochondria, leading to delayed aging and improved cellular function. SC’s specific regulation of inflammatory signaling and vascular homeostasis directly addresses the aging hallmark of “inflammaging.” Research indicates that SC exerts its effects by inhibiting key pro-inflammatory signaling pathways such as PI3K/Akt, MAPK, and NF-κB, while activating endogenous antioxidant defenses, primarily through the Nrf2/ARE pathway. Additionally, SC fine-tunes immune responses by promoting the polarization of microglia/macrophages from a pro-inflammatory M1 phenotype to an anti-inflammatory M2 phenotype, a process regulated via the MAPK pathways [36,37]. Thus, Com1 forms a comprehensive regulatory circuit, from inhibiting core inflammatory signaling (e.g., TNF) and preserving cellular functions (autophagy and mitochondrial homeostasis) to enhancing tissue perfusion and remodeling the immune microenvironment, maximizing the attenuation of aging-related functional decline. The synergy of Com2 (Rg2, Rk1, OD) is characterized by coordinated control over cell fate decisions (proliferation, apoptosis, senescence) and organelle function (mitochondria). The shared core targets, such as BAX and AKT1, suggest that their synergistic action converges on key regulatory hubs for apoptosis and survival signal integration. Rk1, as a specialist in cell cycle and apoptosis regulation, targets the AMPK and mTOR pathways, providing a direct “switch” to intervene in cellular senescence processes [36,38]. Rg2 continues to exert its specific role in autophagy and metabolism, ensuring the timely clearance of aberrant proteins and damaged organelles [35]. Ophiopogonin D likely exerts its anti-aging effect by specifically inhibiting ribosomal protein L22 (RPL22), as indicated by its high binding affinity to RPL22 (−9.41 kcal/mol), identified through virtual screening [39]. For Com3 (Rg2, OD, SA), the three compounds share five core targets and are enriched in MAPK, p53, TNF, autophagy, and cellular senescence pathways. Consistent with its role in other combinations, Rg2 precisely regulates autophagy and glucose metabolism, while OD focuses on mitochondrial function and microenvironmental stability. Extensive experimental evidence supports SA’s protective effects, primarily through the activation of antioxidant signaling pathways such as Nrf2/HO-1 [40], inhibition of inflammasome activation (e.g., NLRP3) [41], regulation of ferroptosis [42], and modulation of critical signaling cascades, including Wnt/*β*-catenin and PI3K/AKT [43]. These mechanisms form the pharmacological basis for SA’s anti-aging properties. The synergistic mode of Com3 combines the specific regulation of autophagy and mitochondrial function with comprehensive modulation of signal transduction, thereby enhancing its overall anti-aging effect. Collectively, the three superior combinations exert synergistic anti-aging effects through a conserved regulatory mode targeting the “signal transduction – cellular homeostasis - stress response” axis. By regulating core aging-related pathways and their key targets, the combinations alleviate oxidative stress, inhibit cellular senescence, restore metabolic homeostasis, and ameliorate inflammaging, ultimately delaying the aging process. The differences in the regulatory networks of the three combinations are primarily reflected in the specific targets and pathway preferences of each compound, which provides a basis for selecting the optimal combination based on distinct aging-related pathological states.

From a theoretical standpoint, this study enriches the framework of AI-driven TCM research by establishing a multi-dimensional feature fusion model for synergistic compound prediction. It bridges the gap between computational predictions and experimental validation, presenting a novel paradigm for the modernization of TCM. In practical applications, the identified synergistic combinations hold potential for development as novel anti-aging drugs, particularly for the prevention and treatment of age-related neurodegenerative diseases. For policymakers, this study highlights the value of integrating AI technology with traditional medicine research, advocating for increased investment in this interdisciplinary field to foster the innovation and industrialization of TCM.

Despite these significant findings, several limitations of this study should be acknowledged, and the evidence presented should be interpreted within an exploratory, proof-of-concept framework. First, although DeepSMCA demonstrated robust internal cross-validation performance, external validation on an independent dataset (derived from Geroprotectors.org and DrugCentral, n = 85) yielded a moderate AUC of 0.626 using the dual-modality (D1 + D2) submodel, because the PPI modality-the largest contributor to the model’s discriminative signal-was not constructible for most external compounds. This performance gap reflects modality degradation, cross-database domain shift, and label noise inherent to the closed-world assumption on negative controls. Consequently, claims regarding model generalizability should be interpreted within an exploratory, proof-of-concept framework, and future work will prioritize constructing multi-modal features for external compounds and incorporating structurally more diverse training data to mitigate domain shift.

Beyond these data-related constraints, three aspects of model generalizability merit explicit discussion. The first concerns potential overfitting to the training data distribution: although five-fold cross-validation and the stable plateau of validation accuracy (approximately 0.96) argue against severe overfitting, the training corpus (914 compounds) is inevitably enriched in well-studied chemical classes, and the marked performance gap between internal (AUC = 0.9849) and external (AUC = 0.626) validation indicates that predictive accuracy remains partially dependent on the training distribution, such that expanding the training set to encompass more diverse chemotypes remains a priority. A second concern is the sensitivity of the model to ADMET prediction errors: the ADMET parameters constituting the D2 feature dimension are themselves computational predictions obtained from ADMETlab, and inaccuracies at this level may propagate into the integrated feature representation and bias classification outcomes; given that ADMET features accounted for 17.1% of total SHAP importance and that several of them (e.g., NR-ER-LBD, MDCK, and CYP1A2-inh) ranked among the most influential individual predictors, future work will incorporate experimentally measured pharmacokinetic data and uncertainty-aware feature weighting to quantify and mitigate this sensitivity. Finally, because the present validation was confined to a single formulation (DZSM), the extent to which DeepSMCA generalizes to compounds with different chemical scaffolds and to other TCM formulas remains to be established, and prospective benchmarking on structurally diverse scaffold libraries and additional formulations will be essential to confirm broader applicability.

Second, because DeepSMCA operates on predicted probabilities and dose–response gradients were not available, the comparison of synergy metrics was performed using probability-level adaptations of the Loewe and HSA null hypotheses rather than their classical dose–response-based formulations; future work incorporating experimentally measured dose–response matrices will enable direct benchmarking against classical Loewe additivity and ZIP models. Accordingly, the proposed “signal transduction–cellular homeostasis–stress response” regulatory axis should be regarded as a hypothesis-generating framework requiring causal validation through targeted genetic and pharmacological experiments.

Third, the HPLC-MS analysis was designed for qualitative profiling; absolute quantification of individual constituents was not performed, and the in vitro validation employed equimolar ratios that do not replicate the natural stoichiometry of the formulation. Future studies will quantify Com1-3 constituents in DZSM and optimize dose ratios reflecting their natural proportions *in vivo*.

## Conclusion

In conclusion, this study successfully developed the DeepSMCA model for identifying synergistic anti-aging compound combinations from DZSM formulation. The findings not only reveal the material basis and molecular mechanisms behind DZSM’s anti-aging effects but also provide a novel strategy for discovering anti-aging drugs from TCM formulations. This interdisciplinary research, combining AI and TCM, holds great promise for advancing the modernization and internationalization of TCM while addressing the global challenge of aging.

## Supporting information

S1 Table Parameters of molecular descriptors. (DOC)

S2 Table Parameters of ADMET. (DOC)

S3 Table Top ten superior combinations predicted by DeepSMCA. (DOC)
