# Supplementary material for: Artificial intelligence-driven identification and mechanistic exploration of synergistic anti-aging compounds from Dengzhan Shengmai formulation
Source: PLoS One. 2026 Sep 8;21(9):e0356888. doi: 10.1371/journal.pone.0356888 (PMC13552753; doi:10.1371/journal.pone.0356888)
Supplement: Supplementary file 1 [file pone.0356888.s001.doc]

**S1 Table. Parameters of molecular descriptors**

|  | MaxEStateIndex | MinEStateIndex | MaxAbsEStateIndex | MinAbsEStateIndex |
| --- | --- | --- | --- | --- |
|  | HeavyAtomMolWt | ExactMolWt | NumValenceElectrons | NumRadicalElectrons |
|  | MaxAbsPartialCharge | MinAbsPartialCharge | FpDensityMorgan1 | FpDensityMorgan2 |
|  | BCUT2D_MWLOW | BCUT2DCHGHI | BCUT2D_CHGLO | BCUT2D_LOGPHI |
|  | BCUT2D_MRLOW | BalabanJ | BertzCT | Chi0 |
|  | Chi1 | Chi1n | Chi1v | Chi2n |
|  | Chi3v | Chi4n | Chi4v | Chi2n |
|  | MaxPartialCharge | FpDensityMorgan3 | BCUT2DLOGPLOW | Chi0n |
|  | Chi2v | HallKierAlpha | Ipc | MolWt |
|  | MinPartialCharge | BCUT2D_MWHI | BCUT2D_MRHI | Chi0v |
|  | Kappa2 | Kappa3 | LabuteASA | Chi3n |
|  | PEOE_VSA12 | PEOE_VSA13 | PEOE_VSA1 | Kappa1 |
| PEOE_VSA2 | | PEOE_VSA3 | PEOE_VSA10 | PEOE_VSA14 |
|  | PEOE_VSA4 | PEOE_VSA5 | PEOE_VSA11 | PEOE_VSA6 |
|  | NumAromaticHeterocycles | PEOE_VSA8 | PEOE_VSA9 | SMR_VSA1 |
|  | NumAromaticRings | SMR_VSA7 | SlogP_VSA12 | SlogP_VSA7 |
|  | NumHAcceptors | SMR_VSA2 | SMR_VSA3 | SMR_VSA4 |
|  | NumHDonors | SMR_VSA8 | SMR_VSA9 | SlogP_VSA1 |
|  | NumHeteroatoms | SlogP_VSA2 | SlogP_VSA3 | SlogP_VSA4 |
|  | NumRotatableBonds | SlogP_VSA8 | SlogP_VSA9 | TPSA |
|  | NumAliphaticHeterocycles | EState_VSA2 | EState_VSA3 | EState_VSA4 |
|  | NumAliphaticRings | EState_VSA8 | EState_VSA9 | VSA_EState1 |
|  | NumAromaticCarbocycles | SMR_VSA6 | NHOHCount | SMR_VSA5 |
|  | NumSaturatedCarbocycles | SlogP_VSA11 | NOCount | SlogP_VSA10 |
|  | NumSaturatedHeterocycles | SlogP_VSA6 | SlogP_VSA5 | PEOE_VSA7 |
|  | NumSaturatedRings | EState_VSA10 | EState_VSA1 | SMR_VSA10 |
|  | fr_Ndealkylation1 | EState_VSA6 | EState_VSA5 | EState_VSA11 |
|  | fr_Al_OH_noTert | VSA_EState2 | VSA_EState10 | EState_VSA7 |
|  | NumAliphaticCarbocycles | VSA_EState8 | VSA_EState7 | VSA_EState9 |
|  | fr_ArN | VSA_EState6 | VSA_EState4 | Ring Count |
|  | fr_COO2 | HeavyAtomCount | FractionCSP3 | MolLogP |
|  | fr_NH0 | VSA_EState3 | VSA_EState5 | MolMR |
|  | fr_Nhpyrrole | fr_Al_COO | fr_Al_OH | fr_COO |
|  | fr_amide | fr_Ar_COO | fr_Ar_NH | fr_Imine |
|  | fr_barbitur | fr_C_O | fr_C_S | fr_Ndealkylation2 |
|  | fr_epoxide | fr_NH1 | fr_Ar_OH | fr_azide |
|  | fr_hdrzine | fr_SH | fr_HOCCN | fr_allylic_oxid |
|  | fr_benzene | fr_amidine | fr_N_O | fr_azo |
|  | fr_dihydropyridin | fr_Ar_N | fr_aryl_methyl | fr_halogen |
|  | fr_isothiocyan | fr_C_O_noCOO | fr_bicyclic | fr_morpholine |
|  | fr_phenol_noOrthoHbond | fr_NH2 | fr_furan | fr_oxazole |
|  | fr_nitro_arom_nonortho | fr_aldehyde | fr_imide | fr_diazo |
|  | fr_alkyl_carbamate | fr_alkyl_halide | fr_lactone | fr_guanido |
|  | fr_benzodiazepine | fr_ketone_Topliss | fr_aniline | fr_isocyan |
|  | fr_para_hydroxylation | fr_piperzine | fr_ester | fr_ether |
|  | fr_term_acetylene | fr_sulfonamd | fr_hdrzone | fr_imidazole |
|  | fr_prisulfonamd | fr_ketone | fr_oxime | fr_sulfide |
|  | fr_unbrch_alkane | fr_nitrile | fr_piperdine | fr_thiocyan |
|  | fr_nitro_arom | fr_nitro | fr_priamide | fr_pyridine |
|  | fr_phos_ester | fr_thiophene | fr_sulfone | fr_tetrazole |
|  | fr_phos_acid | fr_lactam | fr_methoxy | fr_urea |
|  | fr_thiazole | fr_phenol | fr_nitroso | fr_quatN |
