# Supplementary material for: Artificial intelligence-driven identification and mechanistic exploration of synergistic anti-aging compounds from Dengzhan Shengmai formulation
Source: PLoS One. 2026 Sep 8;21(9):e0356888. doi: 10.1371/journal.pone.0356888 (PMC13552753; doi:10.1371/journal.pone.0356888)
Supplement: Supplementary file 2 [file pone.0356888.s002.doc]

**S2 Table. Parameters of ADME**

| **Absorption** | **Distribution** | **Metabolism** | **Excretion** |
| --- | --- | --- | --- |
| Caco-2 Permeability | PPB | CYP1A2 inhibitor | CL |
| MDCK Permeability | VD | CYP1A2 substrate | T_1/2_ |
| Pgp-inhibitor | BBB Penetration | CYP2C19 inhibitor |  |
| Pgp-substrate | Fu | CYP2C19 substrate |  |
| HIA |  | CYP2C9 inhibitor |  |
| F_20%_ |  | CYP2C9 substrate |  |
| F_30%_ |  | CYP2D6 inhibitor |  |
|  |  | CYP2D6 substrate |  |
|  |  | CYP3A4 inhibitor |  |
|  |  | CYP3A4 substrate |  |
