# Supplementary material for: Artificial intelligence-driven identification and mechanistic exploration of synergistic anti-aging compounds from Dengzhan Shengmai formulation
Source: PLoS One. 2026 Sep 8;21(9):e0356888. doi: 10.1371/journal.pone.0356888 (PMC13552753; doi:10.1371/journal.pone.0356888)
Supplement: Supplementary file 3 [file pone.0356888.s003.doc]

**S3 Table. Top ten superior combinations prediction of DeepSMCA**

| **NO.** | **Superior Combinations** | **S_c_ (%)** |
| --- | --- | --- |
| 1 | Ginsenoside Rg1, Ginsenoside Rg2, Scutellarin | 99.99 |
| 2 | Ginsenoside Rg2, Ginsenoside Rk1, Ophiopogonin D | 99.99 |
| 3 | Ginsenoside Rg2, Ophiopogonin D, Schisandrin A | 99.99 |
| 4 | Ginsenoside Rg1, Ginsenoside Rk1, Ophiopogonin D | 99.93 |
| 5 | Ginsenoside Ro, Ginsenoside Rb1, Ergosterol | 99.60 |
| 6 | Ginsenoside Rg2, Ginsenoside Ro, Ergosterol | 99.15 |
| 7 | Ginsenoside Ro, Ginsenoside Rb1, 4-Caffeoyldihydrocaffeic acid | 98.97 |
| 8 | Ginsenoside Rb1, Ginsenoside Rh4, Ophiopogonin D | 98.32 |
| 9 | Ginsenoside Rg1, Ginsenoside Ra1, Ginsenoside f1 | 98.21 |
| 10 | Ginsenoside Rb1 , Ginsenoside Ra1, Ergosterol | 98.18 |
